# Exploring the Allosteric Mechanism of Src Homology-2 Domain-Containing Protein Tyrosine Phosphatase 2 (SHP2) by Molecular Dynamics Simulations

**DOI:** 10.3389/fchem.2020.597495

**Published:** 2020-11-23

**Authors:** Quan Wang, Wen-Cheng Zhao, Xue-Qi Fu, Qing-Chuan Zheng

**Affiliations:** ^1^Edmond H. Fischer Signal Transduction Laboratory, College of Life Sciences, Jilin University, Changchun, China; ^2^Laboratory of Theoretical and Computational Chemistry, International Joint Research Laboratory of Nano-Micro Architecture Chemistry, Institute of Theoretical Chemistry, Jilin University, Changchun, China

**Keywords:** SHP2, PD-1, MD simulations, allosteric, PCA, motion modes

## Abstract

The Src homology-2 (SH2) domain-containing protein tyrosine phosphatase 2 (SHP2, encoded by *PTPN11*) is a critical allosteric phosphatase for many signaling pathways. Programmed cell death 1 (PD-1) could be phosphorylated at its immunoreceptor tyrosine-based inhibitory motif (ITIM) and immunoreceptor tyrosine-based switch motif (ITSM) and can bind to SHP2 to initiate T cell inactivation. Although the interaction of SHP2-PD-1 plays an important role in the immune process, the complex structure and the allosteric regulation mechanism remain unknown. In this study, molecular dynamics (MD) simulations were performed to study the binding details of SHP2 and PD-1, and explore the allosteric regulation mechanism of SHP2. The results show that ITIM has a preference to bind to the N-SH2 domain and ITSM has almost the same binding affinity to the N-SH2 and C-SH2 domain. Only when ITIM binds to the N-SH2 domain and ITSM binds to the C-SH2 domain can the full activation of SHP2 be obtained. The binding of ITIM and ITSM could change the motion mode of SHP2 and switch it to the activated state.

## Introduction

Protein tyrosine phosphorylation is a common post-translational modification, which plays an important role in cellular signaling pathways. Protein tyrosine phosphatases (PTPs) are in charge of removing the phosphate groups, accompanied by protein tyrosine kinases (PTKs), which adjust the homeostasis of tyrosine phosphorylation in cell. Dysregulation of phosphorylation and dephosphorylation can lead to various human diseases such as cancer (Cohen, 2000). In recent decades, PTKs have been successfully targeted many times to treat diseases (Bhullar et al., 2018). But for PTPs, there are still much confusion and many challenges (Tonks, 2013; Fahs et al., 2016).

**Figure 1:**
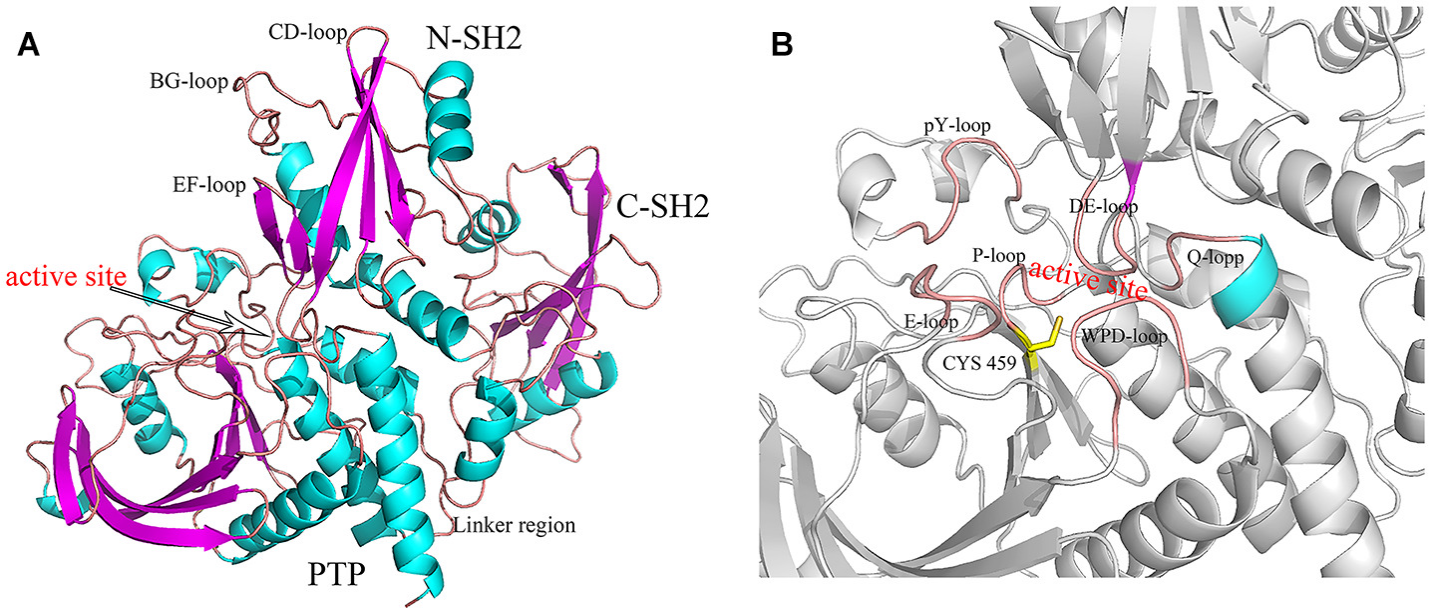
The structure of SHP2 **(A)** and the active site **(B)**. The key regions were marked.

The Src homology-2 (SH2) domain-containing protein tyrosine phosphatase 2 (SHP2, encoded by *PTPN11*) has been one of the hottest topics in the world and has attracted much attention to study the possibility in cancer (Mohi and Neel, 2007; Chan et al., 2008). SHP2 is composed of two SH2 domains (N-SH2, C-SH2), a PTP domain (Figure 1) and a disordered C-terminal tail with two phosphorylation sites (Y542 and Y584). In most studies, SH2 and PTP domains are necessary for the functions of SHP2 (Neel et al., 2003a,b; Pao et al., 2007). The C-terminal tail tyrosine could be phosphorylated in some signaling pathways, and plays a putative regulatory function (Neel et al., 2003b; Marasco et al., 2020). SHP2 has a unique autoinhibited mechanism. In its basal state, the activity of SHP2 is suppressed by intramolecular interactions between residues in the DE loop of the N-SH2 domain and the active site of the PTP domain (Figure 1B) (Barford and Neel, 1998; Hof et al., 1998). However, the phosphotyrosine (pY) peptides binding to the N-SH2 domains induce the conformation rearrangement and disrupt the autoinhibitory face (Barford and Neel, 1998; Hof et al., 1998). In other words, SHP2 is an allosteric enzyme. In fact, both the N-SH2 and C-SH2 domains have the phosphopeptides binding site. The bidentate phosphopeptides (containing two phosphotyrosine) can bind to N-SH2 and C-SH2 simultaneously and activate SHP2 stronger than the mono-phosphopeptide (Wandless et al., 1995; Marasco et al., 2020).

Programmed cell death 1 (PD-1) is a negative costimulatory receptor that is expressed by all T cells during activation. PD-1 regulates T cell effector functions in physiological responses and becomes the paradigm for study the diverse physiological function of inhibitory receptors (Sharpe and Pauken, 2018). PD-1 belongs to the immunoglobulin superfamily and contains two phosphotyrosine motifs in its cytoplasmic tail, namely immunoreceptor tyrosine-based inhibition motif (ITIM) and immunoreceptor tyrosine-based switch motif (ITSM) (Okazaki et al., 2001). Phosphorylated ITIM and ITSM recruit and activate SHP2 and induce down-stream signaling (Okazaki et al., 2001). Ultimately, it leads to negative regulation of both cytokine production and T cell-mediated immune response (Hui et al., 2017). The success of monoclonal antibodies nivolumab and pembrolizumab suggests that blocking the SHP2-PD-1 interaction could be an efficient method of carrying out anticancer therapy (Domling and Holak, 2014; Brahmer et al., 2015). Although many researchers have paid attention to this (Okazaki et al., 2001; Yokosuka et al., 2012; Hui et al., 2017), the atomic details of the interaction of SHP2-PD-1 are still unknown.

In this study, molecular dynamics (MD) simulations were employed to explore the binding details and allosteric regulation mechanism of SHP2 and PD-1. In this article, we mainly focus on three points: (1) how the phosphorylated ITIM and ITSM combine with SHP2. (2) Is there a correspondence between phosphorylated ITIM and ITSM and two SH2 domains? (3) Explore the allosteric regulation mechanism between SHP2 and PD1. Our work may provide useful information for explaining the SHP2 allosteric mechanism and the development of anticancer therapy.

## Methods

### Model Preparation

In this study, we utilized protein-peptides docking to construct the different complex systems by software Discovery Studio (Biovia, 2017). We chose SHP2 crystal structure (PDB code: 4DGP) (Yu et al., 2013) as the initial structure, and adjusted its EF loop according to the crystal structure of SH2 domain and phosphorylated PD-1 complexes (PDB code: 6R5G, 6ROZ, and 6ROY) (Marasco et al., 2020). The reason for adjusting the EF loop is to have enough space to accommodate the phosphorylated PD-1. The details of the docking site were obtained from the co-crystallization of the SH2 domain and phosphorylated PD-1 (PDB code: 6R5G, 6ROZ, and 6ROY). ZDOCK (Chen et al., 2003) was used to obtain the initial docking poses, and RDOCK (Li et al., 2003) was used to optimize and choose the final docking poses.

### MD Simulations

The crystal structures of protein SHP2 [PDB code 4DGP (Yu et al., 2013)] and phosphorylated peptides ITIM [sequence VDpYGELDFD, PDB code:6ROY (Marasco et al., 2020)] and ITSM [sequence EQTEpYATIVFP, PDB code 6R5G (Marasco et al., 2020)] were taken from the Protein Data Bank. The missing residues were complemented by soft MODELER (Sali and Blundell, 1993). In this study, six systems were constructed: SHP2 without ligand (system SHP2); the complex of ITIM and ITSM binding to SHP2 (ITIM bind to N-SH2 domain and ITSM bind to C-SH2 domain, system DUAL); the complex of ITIM binding to N-SH2 domain (system N-SH2-ITIM); the complex of ITSM binding to N-SH2 domain (system N-SH2-ITSM); the complex of ITIM binding to C-SH2 domain (system C-SH2-ITIM) and the complex of ITSM binding to C-SH2-domian (system C-SH2-ITSM). The C tail of SHP2 was removed because it is disordered and unable to obtain the crystal structure. Unless otherwise specified, ITIM and ITSM always indicate the phosphorylated form of the motifs. The residues number of ITIM and ITSM have been renumbered, the phosphotyrosines were designated as residues zero. The residues on phosphopeptides will be identified by one letter (except the phosphotyrosine, PTR), and the residues on SHP2 will be identified by three letters to distinguish them. The structures of the complex of SHP2 and phosphopeptides (ITIM and ITSM) were obtained by soft Discovery Studio (Biovia, 2017) according previous studies (Hayashi et al., 2017; Marasco et al., 2020). The protonation of all systems were assigned based the results of H++ online website (Gordon et al., 2005). MD simulations were performed by AMBER16 software package (Case et al., 2016a) with the classical force-field ff14SB force field (Maier et al., 2015). The force field of phosphotyrosines was obtain from the AMBER parameter database (Khoury et al., 2013, 2014). Sodium ions (Na^+^) and chloride ions (Cl^−^) were added to keep the whole system in an electric neutral state by t-LEaP module (Case et al., 2016b). All systems were solvated with the TTP3P water model in a truncated octahedron with a 10 Å cutoff between the proteins and box boundary under the simulations (Jorgensen et al., 1983). The complex structures were initially fixed with a 100 kcal mol^−1^ Å^−2^ constraint and minimized the energy of water and ions for 10,000 steps of steepest descent (SD) method and 12,000 steps of conjugate gradient (CG) algorithms. Subsequently, the minimization was repeat for 10,000 steps of SD and 8,000 steps of CG without restraints. Thereafter the temperature was increase gently to 310 K with restraints by a 10 kcal mol^−1^ Å^−2^ on the solute atoms and then equilibrated for 5 ns (Uberuaga et al., 2004) Finally, 1,000 ns MD simulations were performed for every system to get the MD simulations trajectories. Particle-Mesh Ewald (Darden et al., 1993) technique was used with a non-bonded cutoff of 12 Å to limit the direct space sum to treat the long range electrostatic interactions. All bonds involving hydrogen atoms were held fixed using SHAKE algorithm (Ryckaert et al., 1977). In this study, all visualization of the structures and trajectories were done by software package VMD (Humphrey et al., 1996), Chimera (Pettersen et al., 2004) and PyMOL (DeLano, 2014).

### The Binding Free Energy and Decomposition Analysis

The molecular mechanics Generalized Born Surface Area (MM/GBSA) method (Kollman et al., 2000; Hou et al., 2012; Sun et al., 2014a,b) was performed to calculate the binding free energy of SHP2 and phosphopeptides. The calculation formulas are shown as follows:

(1) $$\begin{array}{r} G_{bind}\;=\;G_{complex}-\;\left(G_{receptor}+G_{ligand}\right) \end{array}$$

The ΔG_bind_ represents the total binding free energy. The G_complex_, G_receptor_ and G_ligand_ are the free energy of complex, receptor, and ligand, respectively.

(2) $$\begin{array}{r} G_{bind}\;=\;E_{MM}\;+\;G_{sol}\;-\;TS \end{array}$$

(3) $$\begin{array}{r} E_{MM}\;=\;E_{ele}\;+\;E_{vdw}\;+\;E_{int} \end{array}$$

(4) $$\begin{array}{r} G_{sol}\;=\;G_{PB/GB}\;+\;G_{SA} \end{array}$$

In equation (2), the E_MM_, G_sol_, and TS represent the molecular mechanics component in the gas phase, the stabilization energy due to salvation, and a vibrational entropy term. E_MM_ is the gas phase molecular mechanical energy, G_sol_ is the solvation free energy. E_int_, E_ele_ and E_vdW_ are the internal energy, coulomb energy and van der Waals interaction terms. G_sol_ represents the solvation contribution, and it can be separated into polar solvation energy (ΔG_GB_) and non-polar solvation energy (ΔG_SA_). ΔG_GB_ can be calculated by the Generalized-Boltzmann method (Onufriev et al., 2004). G_SA_ is calculated by:

(5) $$\begin{array}{r} G_{SA}\;=\;\gamma SASA\;+\;\beta \end{array}$$

Here, the γ and β, two empirical constants, were set as 0.0072 kcal mol^−1^Å^−2^ and 0.00 kcal mol^−1^ and SASA is the solvent accessible surface area determined by a probe radius of 1.4 Å. Four thousand snapshots in the last 80 ns trajectories were selected to calculate the binding free energies. The entropy is generally calculated using normal-mode analysis (Weiser et al., 1999) by AMBER 16 software package. One hundred snapshots from the 4,000 snapshots were chosen to calculate the entropy.

To explore the binding mechanism of SHP2 and phosphopeptides, and find the key residues in their binding, the free energy decomposition on a residual basis was performed by MM/GBSA.

### Principal Component Analysis and Free Energy Landscape

Principal component analysis (PCA) (Lauria et al., 2009; Yang et al., 2009) is a widely used method to understand the dynamics of biological systems. PCA involves a mathematical algorithm that reduces the number of pendent motions into a smaller number of independent motions called principal components. The first principal (PC1) is the highest corresponding Eigenvalue which reflects the most important motion under the simulations. In this study, The PCA was performed on the backbone atoms for the last 200 ns trajectories without water and ions. Then, the porcupine plots were generated by ProDy (Bakan et al., 2011) in VMD (Humphrey et al., 1996).

Free energy landscape (FEL) is a useful method to study the allosteric regulation in proteins, and could help us understand the conformation changes related to different energy states (Motlagh et al., 2014; Wodak et al., 2019; Bai, 2020; Bai et al., 2020). In FEL, free energy minima represent stable conformations. The representation of FEL was constructed by PC1 and PC2. The Gibbs free energy (G_i_) is defined as follows:

(6) $$\begin{array}{r} G_{i}\;=\;-k_{B}T\;ln{\left(N_{i}/N_{max}\right)}_{\;} \end{array}$$

In equation (6), *k*_*B*_ is Boltzmann's constant, T is the absolute temperature, *N*_*i*_ is the probability density of the MD data, and *N*_max_ is the maximum probability.

## Results

### The Overall Structural Properties

**Figure 2:**
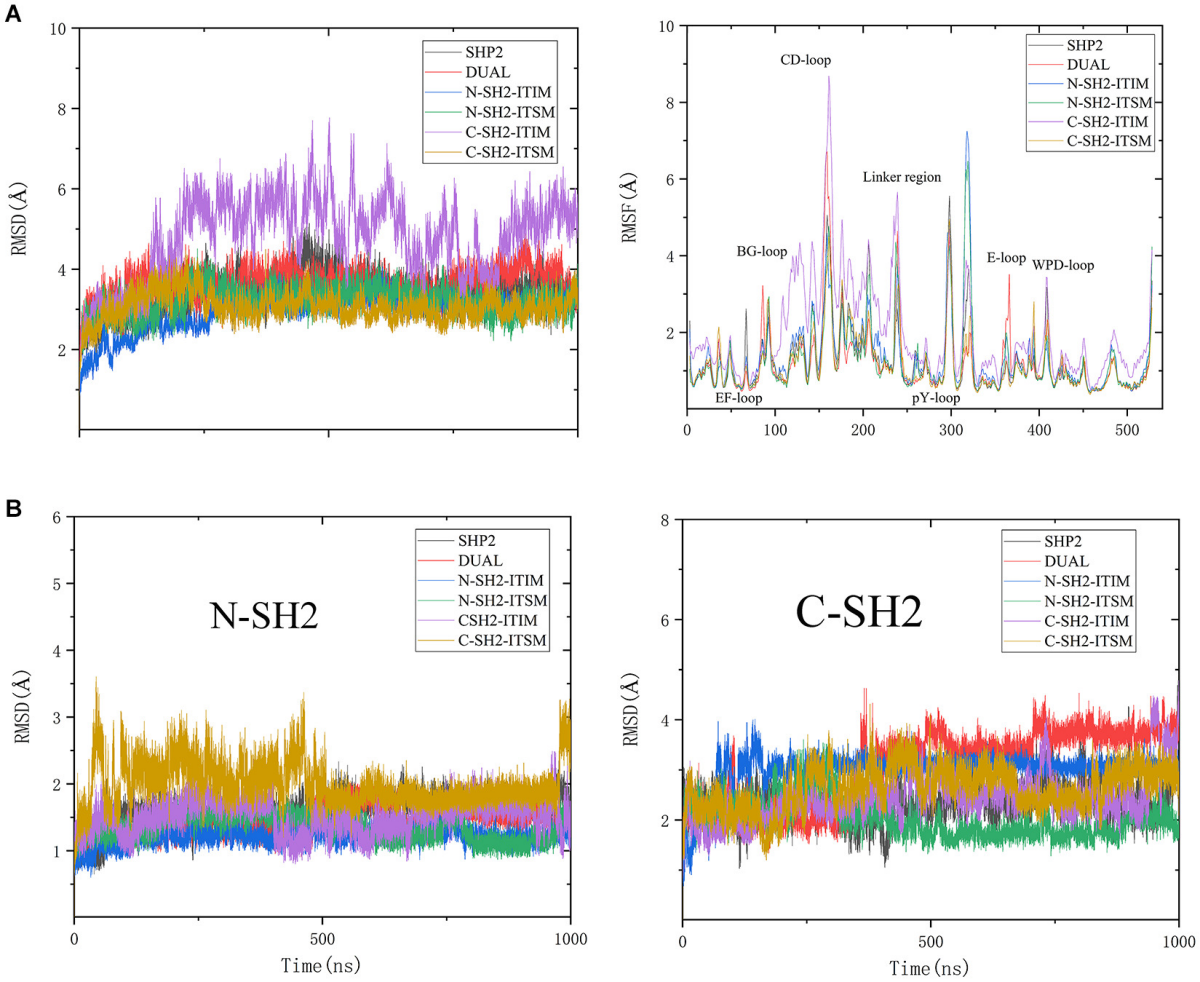
RMSD values and RMSF value of SHP2 in all systems **(A)**, and the RMSD values of N-SH2 and C-SH2 domains in all systems **(B)**.

To get more information on the overall structural changes, root-mean-square deviations (RMSD) and root-mean-square-fluctuations (RMSF) of the backbone atoms that reference to the initial structure were performed (Figure 2). As shown in Figure 2A, the RMSD values of all systems are stable during the whole simulation except for system C-SH2-ITIM. The RMSD curve of system C-SH2-ITIM is more volatile and has higher values than other systems, even the DUAL system. This means that ITIM binding to C-SH2 domain could disturb the stability of SHP2. In addition, the RMSD values of N-SH2 (6-102), C-SH2 (110-216) and PTP (247-517) domains were also calculated separately for all systems due to the particularity of SHP2. The PTP domain of SHP2 could keep a stable conformation in all systems, no matter whether SHP2 binds to ITIM and ITSM or not (Supplementary Figure 1). For the N-SH2 domain, the RMSD values of system C-SH2-ITSM are higher than other systems (Figure 2B). In other words, ITSM binding to C-SH2 disturbs the structure of the N-SH2 domain. For the C-SH2 domain, the difference of each system is more significant, and the RMSD values of systems DUAL and N-SH2-ITIM were slightly higher than other systems. This result proves that ITIM binding to N-SH2 can also affect the structure of the C-SH2 domain. In general, the binding of ITIM or ITSM to SHP2 could significantly affect the flexibility of SHP2. The RMSF curves can reflect the effect of ITIM and ITSM binding to SHP2 more clearly (Figure 2A). For system DUAL, EF loop keeps stable, but BG loop becomes more flexible. In addition, the key E loop have a unique change in DUAL system. The flexibility of the C-SH2-ITIM system is higher than other systems, especially in the C-SH2 domain.

The RMSD and RMSF analysis revealed that ITIM binding to N-SH2 can affect the C-SH2 domain, and ITSM binding to C-SH2 can affect the N-SH2 domain. The binding of phosphopeptides can also affect the flexibility of SHP2's key regions. These interesting results may give us a new perspective to explore the allosteric regulation mechanism.

### Binding Free Energy and Decomposition Analysis

To further explore the interaction between SHP2 and phosphopeptides, MM/GBSA calculation and energy decomposition of each complex system was performed. As shown in Table 1, ITIM prefers to bind to the N-SH2 domain, while ITSM has the same binding affinity for N-SH2 and the C-SH2 domain. The affinity of ITIM and ITSM binding to the N-SH2 domain is almost the same, but the affinity of ITSM binding to the C-SH2 domain is stronger than that of ITIM. Previous study has suggested that ITSM has a stronger binding affinity to the SH2 domain than ITIM (Marasco et al., 2020), and this may be due to the entropy that was ignored. For system DUAL, the binding free energy of ITIM and ITSM were calculated, respectively. In this system, the binding affinity of ITIM and ITSM has been weakened than system N-SH2-ITIM and C-SH2-ITSM. However, the reasons for the weakened affinity of ITIM and ITSM are different. The decrease of the affinity of ITIM was caused by the decrease of enthalpy, and the decrease of the affinity of ITSM was caused by the crease of entropy. This weakening of the affinity may be caused by the structure changes in system DUAL. In short, ITIM has a preference for N-SH2 domain, and ITSM has almost no preference for N-SH2 and C-SH2 domain. But in system DUAL, the affinity of ITIM and ITSM to SH2 domain was weakened due to the structure changes of SHP2.

**Table 1:** Binding free energies (kcal mol^−1^) and its components for a complex system.

	**N-SH2-ITIM**	**C-SH2-ITIM**	**N-SH2-ITSM**	**C-SH2-ITSM**	**DUAL-ITIM**	**DUAL-ITSM**
E_ele_	−560.26 ± 62.33	−150.06 ± 32.73	−502.71 ± 21.89	−305.12 ± 32.22	−377.11 ± 65.30	−43.17 ± 27.35
E_vdw_	−56.65 ± 5.30	−47.22 ± 4.94	−66.35 ± 5.85	−60.83 ± 4.95	−57.72 ± 5.22	−55.16 ± 4.40
G_pb_	540.29 ± 57.14	131.48 ± 30.38	482.57 ± 20.26	287.97 ± 30.02	383.97 ± 58.34	30.42 ± 26.01
G_SA_	−8.79 ± 0.51	−7.12 ± 0.42	−10.14 ± 0.57	−9.37 ± 0.45	−9.16 ± 0.53	−8.03 ± 0.43
G_pol_	−19.96 ± 84.56	−18.58 ± 44.66	−20.14 ± 29.83	−17.15 ± 44.04	6.86 ± 87.57	−12.75 ± 37.74
G_nonpol_	−65.44 ± 5.32	−54.37 ± 4.96	−76.49 ± 5.88	−70.20 ± 4.97	−66.88 ± 5.25	−63.19 ± 4.42
H	−85.41 ± 9.63	−73.52 ± 8.02	−96.86 ± 8.87	−87.35 ± 8.73	−60.02 ± 11.80	−75.94 ± 5.82
TS	−40.14 ± 3.94	−37.04 ± 3.42	−52.97 ± 4.23	−42.44 ± 2.08	−43.68 ± 3.86	−49.01 ± 2.68
ΔG	−45.27 ± 10.40	−36.48 ± 8.72	−43.89 ± 9.83	−44.91 ± 8.97	−16.34 ± 12.42	−26.93 ± 6.41

*G_pol_= E_ele_ + G_pb_. G_nonpol_ = E_vdw_ + G_SA_. G_bind_ = G_nonp_ + G_pb_ – TS*.

**Figure 3:**
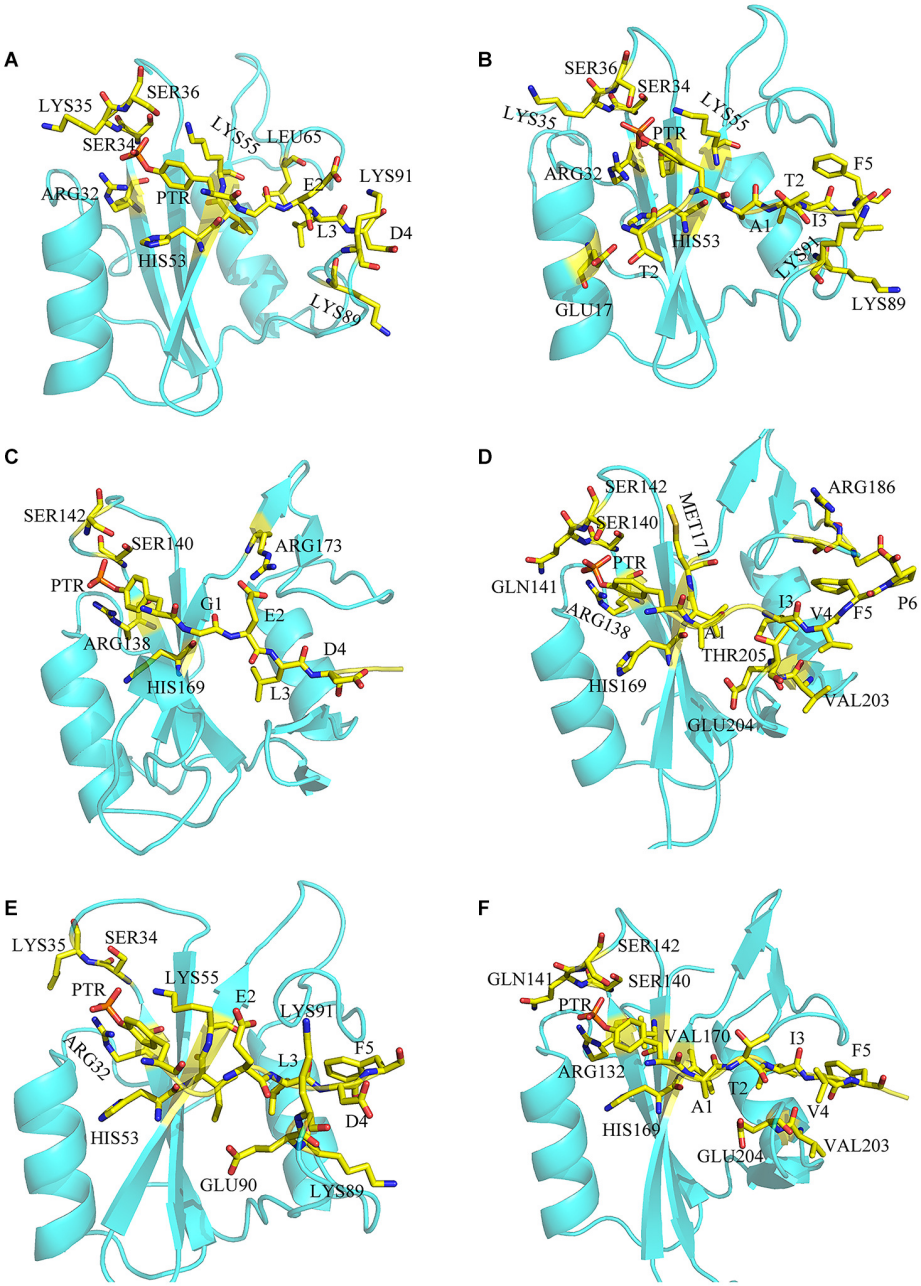
The binding details of systems N-SH2-ITIM **(A)**, N-SH2-ITSM **(B)**, C-SH2-ITIM **(C)**, C-SH2-ITSM **(D)**, and DUAL **(E,F)**.

To have a better understanding of the interaction between SHP2 and phosphopeptides, free energy decomposition on residual basis was performed by MM/GBSA (Sun et al., 2014b) method. Residues with the contribution over −2 kcal mol^−1^ would be discussed emphatically (Supplementary Tables 1–6). For system N-SH2-ITIM (Figure 3A), residues ARG32, SER34, LYS35, SER36, HIS53, LYS55 LYS89 and LYS91 have the largest contribution to the binding due to the electrostatic interaction. Those positive charged residues surround the negative charged PTR, and played a decisive role in the binding. In addition, residues ILE54 and LEU65 also have an important contribution to the binding by van der Waals interaction. For system N-SH2-ITSM (Figure 3B), in addition to the above residues, residues GLU17, THR42 and GLU90 also played an important role in the binding. But the contribution of ILE54 and LEU65 to the binding disappeared.

For system C-SH2-ITIM (Figure 3C), ITIM binds to the C-SH2 domain in the same way that it binds to the N-SH2 domain. Positively charged residues ARG138, ARG173, SER140, SER142, and HIS169 play a major role in the binding. For system C-SH2-ITSM (Figure 3D), there are abundant interactions between ITSM and C-SH2 domain. Residues HIS169, GLY183, ARG186, and THR205 also have key contribution to the binding. Compared with the N-SH2 domain, ITSM has a richer interaction with the C-SH2 domain.

The decomposition of system DUAL was divided into two parts, ITIM binds to N-SH2 (Figure 3E) and ITSM (Figure 3F) binds to C-SH2. Although the contribution of PTR in ITIM has declined, and PTR has almost lost the interaction with SER34, LYS35, and SER36. Binding free energy and decomposition analysis explained the binding mode of the bidentate phosphorylated PD-1 and SHP2, and revealed the selectivity of ITIM and ITSM for the SH2 domain.

### Hydrogen Bond Network Analysis

Hydrogen bond network is an important part of the protein-peptide interaction. We analyzed the hydrogen bond inside the SHP2 and the hydrogen bond between SHP2 and phosphopeptides. For the hydrogen bond inside SHP2, we focused on the hydrogen bond that between N-SH2, C-SH2, and PTP domain. First of all, there is no hydrogen bond interaction between C-SH2 and PTP domain, because of the long linker region (residue 217 to 246) between them. This linker region isolates the hydrogen bond between C-SH2 and PTP domain (Figure 1). There is almost no hydrogen bond interaction between N-SH2 and C-SH2 domain except for system DUAL. For system DUAL, there is a weak hydrogen bond interaction between N-SH2 and C-SH2 domain (GLN176-ARG23, occupancy 53%). There are abundant hydrogen bond interactions between N-SH2 and PTP domain. In system SHP2, several strong hydrogen bonds connected N-SH2 and PTP domain, such as ALA72-GLN506, ASP61-ALA461, ASP61-GLY464, GLY60-GLN510, and ASP61-506 (Supplementary Table 7). The hydrogen bonds between ASP61 and Q-loop (GLN506 to GLN510) should play an important role in the autoinhibition of SHP2. In system DUAL, the binding of ITIM and ITSM weakens the hydrogen bond interactions between ASP61 and Q-loop. Although a new hydrogen bond (GLU76-SER502, occupancy 94%) was formed between N-SH2 and PTP domain (Supplementary Table 8), the stability of the autoinhibition pocket had been broken.

**Figure 4:**
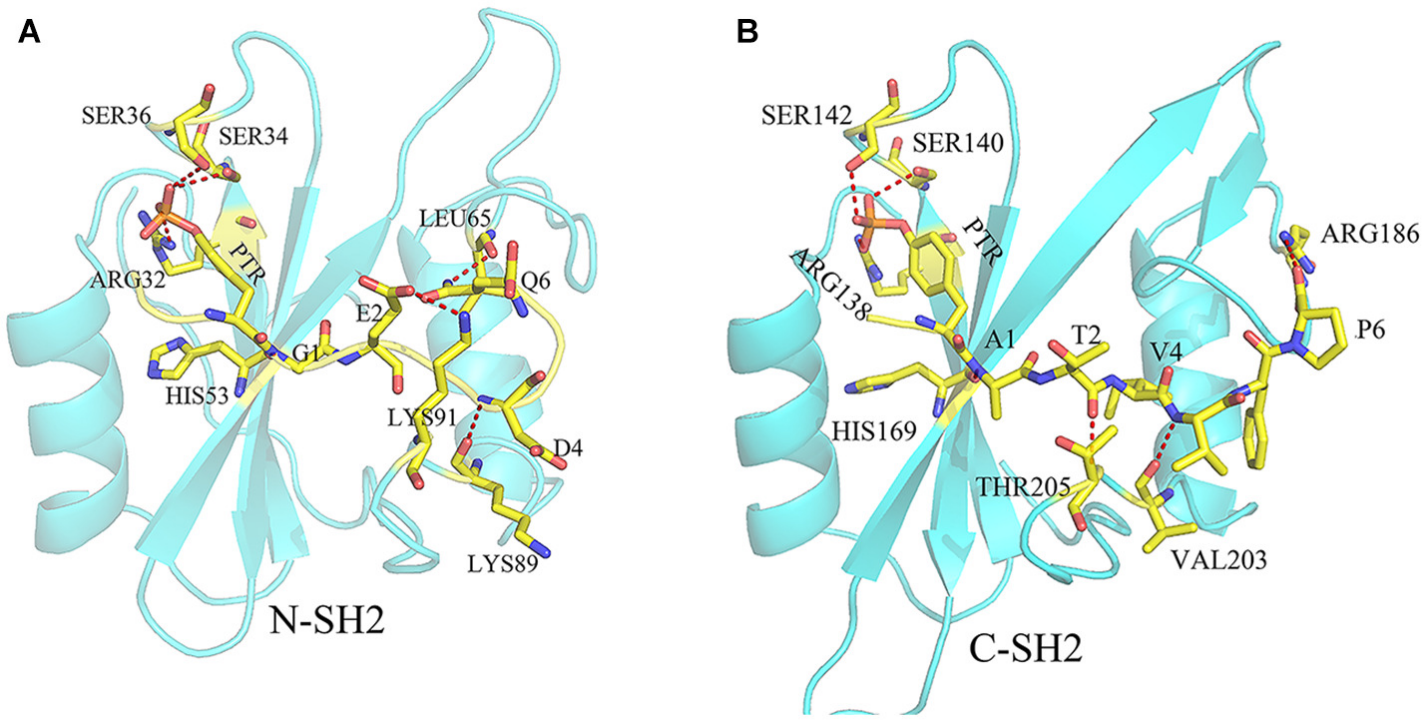
The hydrogen bond frame between ITIM and N-SH2 domain **(A)** and the hydrogen bond frame between ITSM and C-SH2 domain **(B)**. The residues on ITIM or ITSM were identified by one letter (except PTR), and the residues on SHP2 were identified by three letters. The yellow dotted line means there is a hydrogen bond between the two residues.

There are many hydrogen bond interactions between SHP2 and phosphopeptides, and these hydrogen bonds will strengthen the binding of phosphopeptides and SHP2. In systems N-SH2-ITIM (Supplementary Table 9) and N-SH2-ITSM (Supplementary Table 10), N-SH2 domain adopts the same hydrogen bond framework to bind ITIM and ITSM (Figure 4). HIS53 formed a hydrogen bond with residue 1 (G1 in ITIM and A1 in ITSM), LYS89 formed a hydrogen bond with residue 4 (D4 in ITIM and V4 in ITSM), and LYS89 formed hydrogen bond with residue 2 (E2 in ITIM and T2 in ITSM). In addition, the PTR formed a hydrogen bond with ARG32, SER34 and SER36 in these two systems. This hydrogen bond network anchors PTR and its neighboring residues to the binding pocket. The hydrogen bond framework also exists in system C-SH2-ITIM (Supplementary Table 9) and C-SH2-ITSM (Supplementary Table 10). HIS169 formed with residue 1 (G1 in ITIM and A1 in ITSM), VAL203 formed hydrogen bond with residue 4 (D4 in ITIM and V4 in ITSM), THR205 formed hydrogen bond with residue 2 (E2 in ITIM and T2 in ITSM). The PTR could also form hydrogen bonds with ARG138, SER140, and SER142. The hydrogen bond framework also exists in DUAL system (Supplementary Table 11), and the hydrogen bond interactions between C-SH2 and ITSM is stronger than that between N-SH2 and ITIM. The hydrogen bonds between ITIM and ARG32, SER34, and SER36 were disappeared. As shown in the hydrogen bond analysis, ITIM and ITSM binds to SHP2 with a similar hydrogen bond framework. But in system DUAL, the hydrogen bonds between ITIM and N-SH2 domain were weakened, and the hydrogen bonds between N-SH2 and PTP domain were also weakened. These hydrogen bonds changes may be related to the allosteric regulation of SHP2.

### Principal Component Analysis and Porcupine Plots

**Figure 5:**
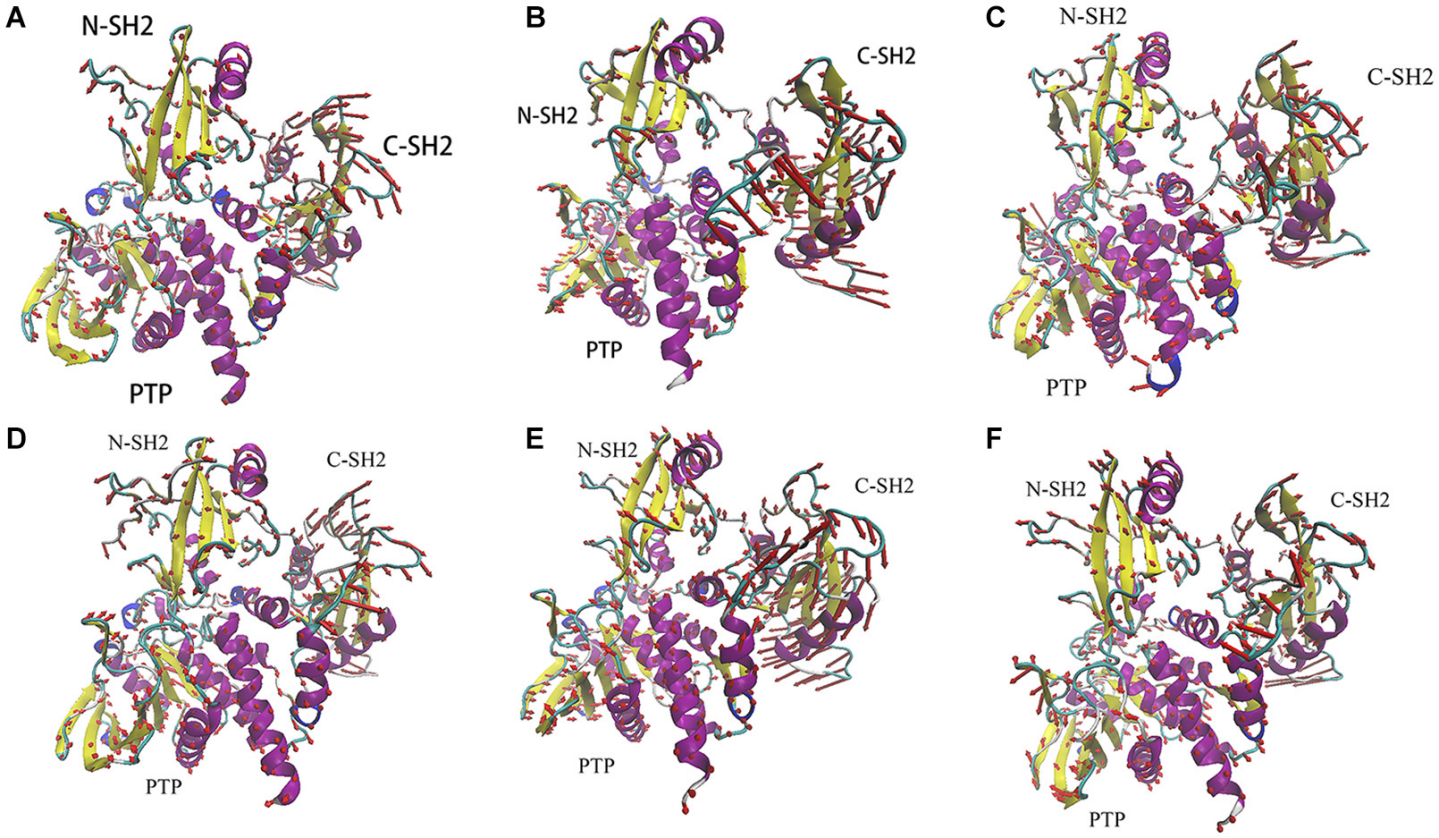
The porcupine plots of system SHP2 **(A)**, DUAL **(B)**, N-SH2-ITIM **(C)**, N-SH2-ITSM **(D)**, C-SH2-ITIM **(E)**, and C-SH2-ITSM **(F)**.

To identify correlated motions of SHP2, we have performed PCA on each system. In this part, we will primarily focus on the principal modes obtained from the highest Eigenvalue (PC1) and the corresponding Eigenvector. It can be well-understood from the porcupine plot figure, which shows the variation in the directions of eigenvectors to the highest Eigenvalue. The relative movement is reflected by the length and direction of the arrows. As shown in Figure 5, the binding of ITIM and ITSM to SHP2 will significantly change the correlated motions in SHP2. When ITIM or ITSM binds to SH2 domain, it not only affects the region where it binds, but also affects the motions of other domains.

Just like the results of RMSD and RMSF analysis, the motion of C-SH2 domain is most obvious. There are different motion modes in different systems. C-SH2 domain generally adopts a clockwise rotation in system SHP2 (Figure 5A), N-SH2-ITSM (Figure 5D), C-SH2-ITIM (Figure 5E), C-SH2-ITSM (Figure 5F), and adopts an anticlockwise rotation in system DUAL (Figure 5B) and N-SH2-ITIM (Figure 5C). This anticlockwise motion mode of C-SH2 domain is probably caused by the binding of ITIM to N-SH2 domain. Combining with experiments research (Marasco et al., 2020), this anticlockwise rotation is beneficial to the activation of SHP2. There are some very complex motion modes in N-SH2 and PTP domain. In this text, we only focus on comparing the system SHP2 (Figure 5A) and DUAL (Figure 5B) to explain the allosteric regulation mechanism. Compared with SHP2 system, the motion modes of N-SH2 domain have changed significantly in system DUAL. The N-SH2 domain flips inward under the driver of ITIM. The key regions in N-SH2, such as FE-loop, BG-loop and DE-loop, also tend to get away from PTP domain. The motion mode of PTP domain is more complicated. E-loop and WPD-loop move outward to open the autoinhibited pocket. P-loop and pY-loop move inward to further release the autoinhibition. In addition, the linker region between C-SH2 and PTP domain which we have overlooked may play a very important role in SHP2 allosteric regulation. In system DUAL, this linker drives the αI and αA to move outward and finally triggers the motion of Q-loop. Moreover, we also found a very interesting phenomenon about this linker: it will maintain the opposite motion modes of C-SH2 (Figure 5). This phenomenon proves that the motion mode of the linker region may be regulated by C-SH2 domain, and further reveals the role of C-SH2 domain in allosteric regulation. In a word, the binding of ITIM and ITSM has completely change the motion modes of SHP2. In system DUAL, its motion modes release the autoinhibited pocket, and promotes SHP2 to the activate state more easily.

It is notable that there is another motion mode (PC2) in systems DUAL, N-SH2-ITIM and N-SH2-ITSM. In system DUAL, the overall trend of these two motion modes (PC1 and PC2) are consistent (activation, Supplementary Figures 2, 3). But in other systems, their PC1 and PC2 represent different motion modes (activation or autoinhibition). In other words, ITIM or ITSM binding to N-SH2 domain can activate SHP2, but ITIM has a stronger activation effect (its PC1 represents the active motion mode). In summary, ITIM and ITSM disturb the equilibrium between the inactive state and active state, and push the equilibrium toward the active state. The porcupine plots of the PC2 of these three systems could be obtained from SI.

### Correlational Analysis

**Figure 6:**
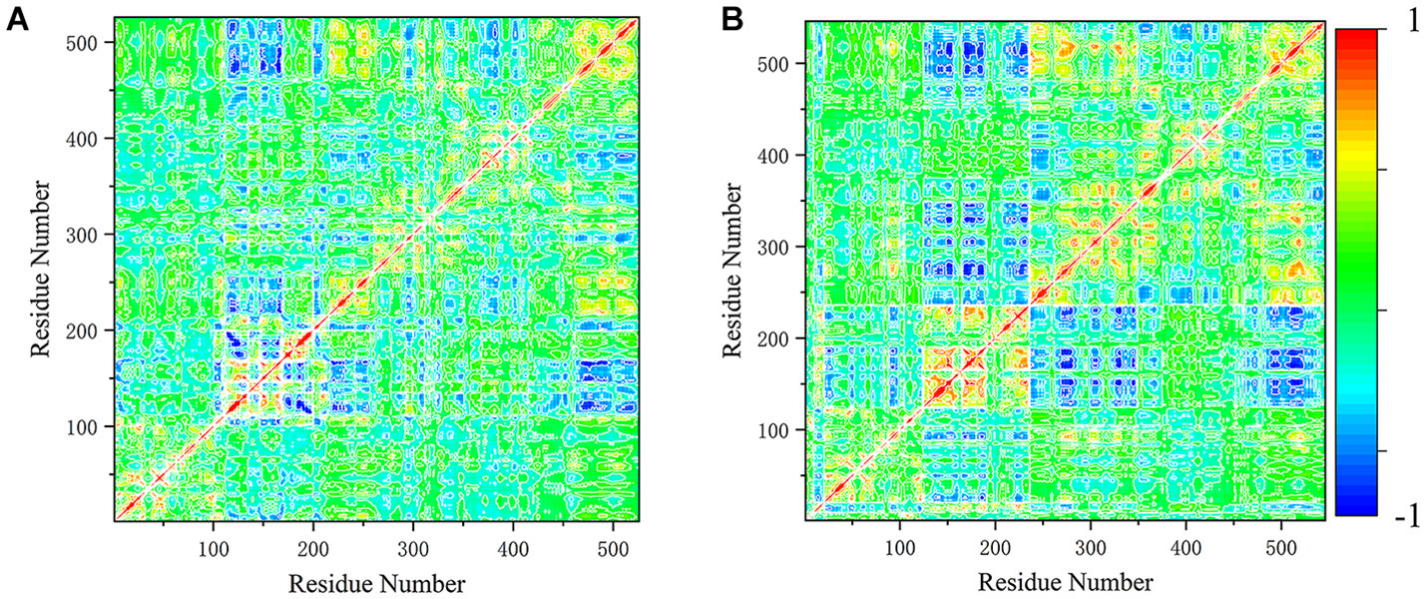
The cross-correlation map of system SHP2 **(A)** and DUAL **(B)**. The covariance values are within the range of −1 to 1, where the extreme positive values (1) reflect the pairs of residues moving in the same direction (red color). And the negative values (−1), where the extreme value reflect those residues moving in opposite direction (blue color).

In order to better understand the differences in the motion modes, these motions were represented quantitatively using cross-correlation map of the C_α_-C_α_ displacement. The positive covariance value reflects the pair of residues moving in the same direction, and the negative value reflects those moving in opposite directions (Figure 6). In system DUAL, the N-SH2 domain moved in the opposite direction to the C-SH2 domain, and the correlation between N-SH2 and other two domains was strengthened. The C-SH2 domain moved in the opposite direction to the linker region and PTP domain. In addition, the positive correlation with C-SH2 and PTP domain was enhanced, which means that these two domains will participate in the allosteric regulation of SHP2 in the form of overall domain. This result indicates the importance of the linker regions and C-SH2 domain, and reveals the rearrangement mechanism of C-SH2 and PTP domain in allosteric regulation.

### ITIM and ITSM Remodel the Free Energy Landscape

**Figure 7:**
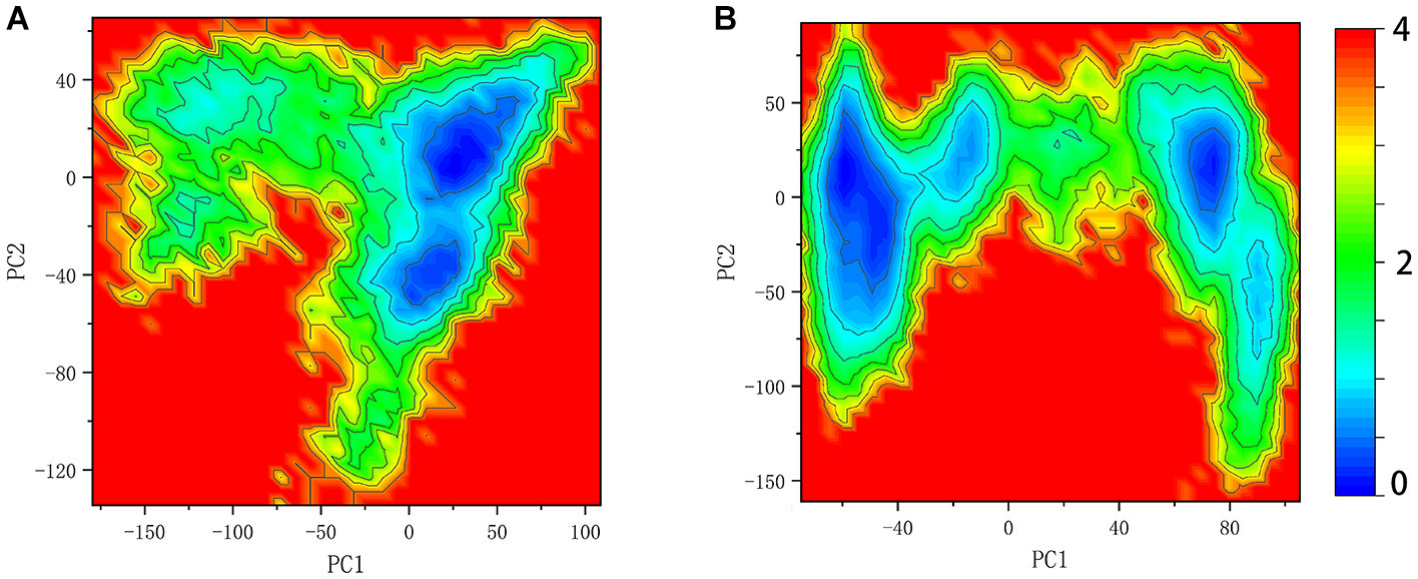
Free energy landscape of system SHP2 **(A)** and DUAL **(B)**.

Here the 2D FEL representation of systems SHP2 and DUAL were performed by PC1 and PC2. As shown in Figure 7, the binding of ITIM and ITSM remodel the free energy landscape. Compared with the SHP2 system, the DUAL system has two main basins, and both of them are helpful to relieve the autoinhibition. System N-SH2-ITIM and N-SH2-ITSM also have two motion modes, but these two modes are “activation” and “inactivation,” respectively (Supplementary Figures 2, 4). System C-SH2-ITIM has two motion modes, and system C-SH2-ITSM has only one motion mode. All motion modes of these two systems are “inactivation.” This result also reveals that only N-SH2 domains bound to ITIM or ITSM could initiate the activation of SHP2.

**Figure 8:**
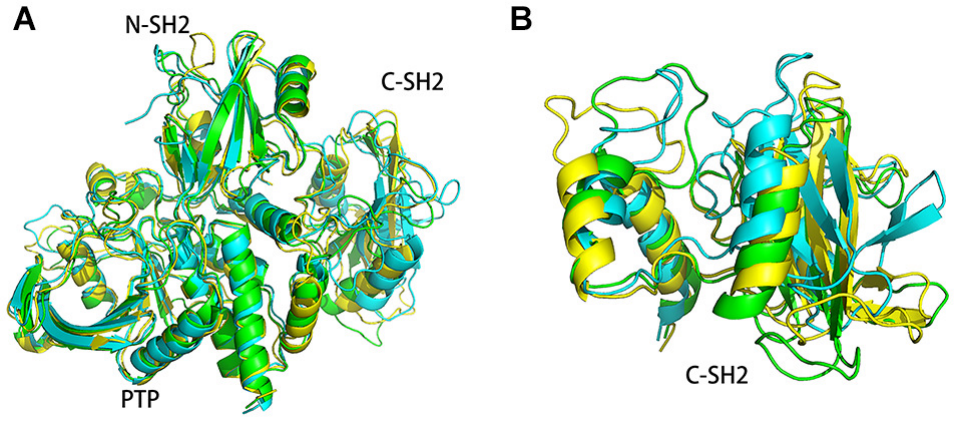
The superimposed structures of system SHP2 (green), DUAL (blue), and the crystal structure (yellow). The superimposed structures of the overall structure **(A)** and C-SH2 domain were shown separately **(B)**.

In order to understand the structure changes more visually, the structures of energy minima in systems SHP2 and DUAL were chosen for further analysis. These structures were superimposed to the crystal structure (4DGP) to compare their structural difference. As shown in Figure 8, there are only slight differences between the SHP2 system and crystal structure. In system SHP2, the BG-loop opens outward, the C-SH2 domain is slightly misplaced. And the linker between C-SH2 and PTP domain has also changed. For system DUAL, the C-SH2 domain has a very obvious rotation (Figure 8B), and this rotation can even change its interface with N-SH2 and PTP domain. The linker between C-SH2 and PTP domain also shifted outward. The changes of C-SH2 and the linker region trigger the change of the helix β (residues 251–261), helix β squeezes inward the autoinhibited pocket. It is very interesting that the superposition of these three structures have an obvious hierarchy. This clear hierarchy further illustrates that the binding of ITIM and ITSM disrupts the equilibrium between the active state and inactivate state of SHP2.

## Discussion

Targeting of SHP2-PD-1 interaction by a drug molecule during immune response is a successful therapeutic strategy for some cancers. The design of small drug molecules targeting the SHP2-PD-1 interface need the knowledge of the structure of the SHP2-PD-1 complex and their allosteric regulation mechanism. In recent years, there have been many efforts on the allosteric mechanism of SHP2. SHP099 is the first successful allosteric inhibitor which binds to the interface of the three domains (actually this binding site is close to the linker between C-SH2 and PTP domains) and anchors them as a “latch” (Chen et al., 2016; Garcia Fortanet et al., 2016). Later, researchers discovered allosteric inhibitors that bind to the interface of N-SH2 and PTP domains, such as SHP244 and SHP844 (Fodor et al., 2018). These inhibitors can be combined with SHP099 to enhance their inhibitory efficacy. These inhibitors essentially prevent the movement of the three domains. Recent study has suggested that the allosteric regulation mechanism of SHP2 is similar to that of SHP1. The C-SH2 domain would rotate 120 degrees to expose the catalytic site (LaRochelle et al., 2018). However, subsequent study has shown that the length of phosphorylated PD-1 does not allow this rotation (Marasco et al., 2020). These studies indicate that the allosteric mechanism of SHP2 is closely related to the movement of the three domains. In this study, we constructed the complex structures of SHP2 (residues 1–526) and phosphorylated PD-1 (ITIM and ITSM). One-thousand ns molecular dynamics (MD) simulations were performed to explored their binding details and allosteric regulation mechanism. The allosteric regulation of SHP2 should be a dynamic state change. SHP2 could move between the activation state and inactivation state spontaneously, and keep an equilibrium. In the natural state, this equilibrium is biased toward the inactivation states. When ITIM and ITSM binds to SHP2, the equilibrium was broken and pushed to the activation state. N-SH2 domain is the key structure to initiate the allosteric regulation, and C-SH2 domain is the important module to activate SHP2. SHP2 will be activated only when ITIM or ITSM bind to the N-SH2 domain, and the binding of ITSM to C-SH2 domain will further push the equilibrium toward the activated state. In addition, the linker region between the C-SH2 domain and PTP domain should play an important role in the allosteric regulation. The binding of phosphorylated PD-1 changes the motion modes of the C-SH2 domain. This linker transmits this change to the PTP domain and weakens the interaction between the N-SH2 domain and the PTP domain. The autoinhibition of SHP2 would be broken. Our study gives the details of the interaction between SHP2 and phosphorylated PD-1, which cannot be obtained by crystallization. We analyzed the motion modes of three domains in different situations, and suggested the motion modes which is conducive to the activation of SHP2. Preventing or reducing these “activation” motion modes may provide a new method for drug discovery.

## Data Availability Statement

The original contributions generated for the study are included in the article/Supplementary Materials, further inquiries can be directed to the corresponding author/s.

## Author Contributions

QW performed experiments, analyzed data, and wrote the paper. W-CZ wrote the supplementary paper. X-QF and Q-CZ designed the experiments and reviewed the manuscript. All authors contribute to the article and approved the submitted version.

## Conflict of Interest

The authors declare that the research was conducted in the absence of any commercial or financial relationships that could be construed as a potential conflict of interest.
