# Supplementary material for: Exploring the Allosteric Mechanism of Src Homology-2 Domain-Containing Protein Tyrosine Phosphatase 2 (SHP2) by Molecular Dynamics Simulations
Source: Front Chem. 2020 Nov 23;8:597495. doi: 10.3389/fchem.2020.597495 (PMC7719740; doi:10.3389/fchem.2020.597495)
Supplement: Supplementary file 1 [file Table_1.DOCX]

Supplementary Material

Figure S1. The RMSD values of PTP domain in all systems.





Figure S2. The PC2 motion modes of systems DUAL (A), N-SH2-ITIM (B) and N-SH2 ITSM (C).


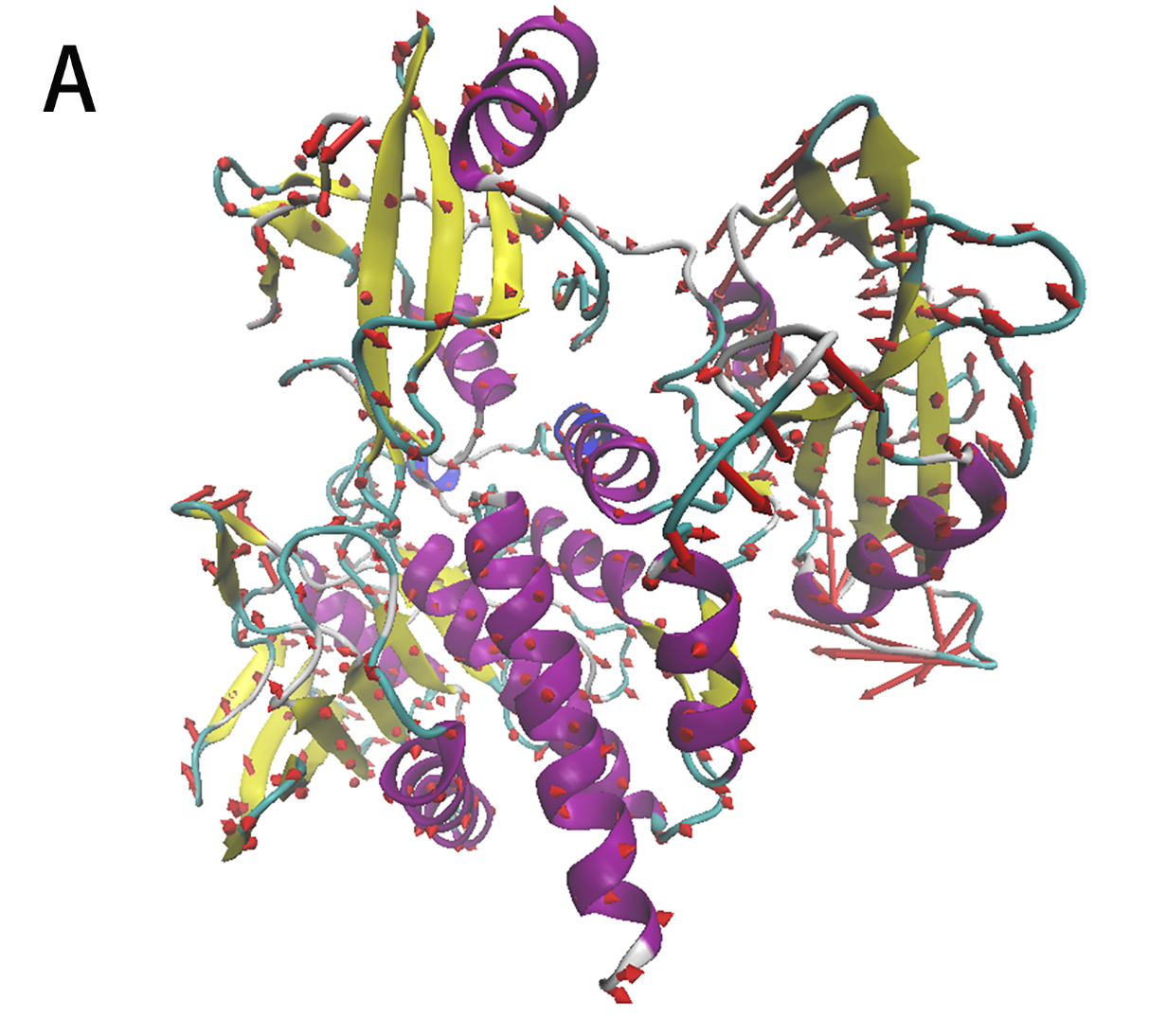


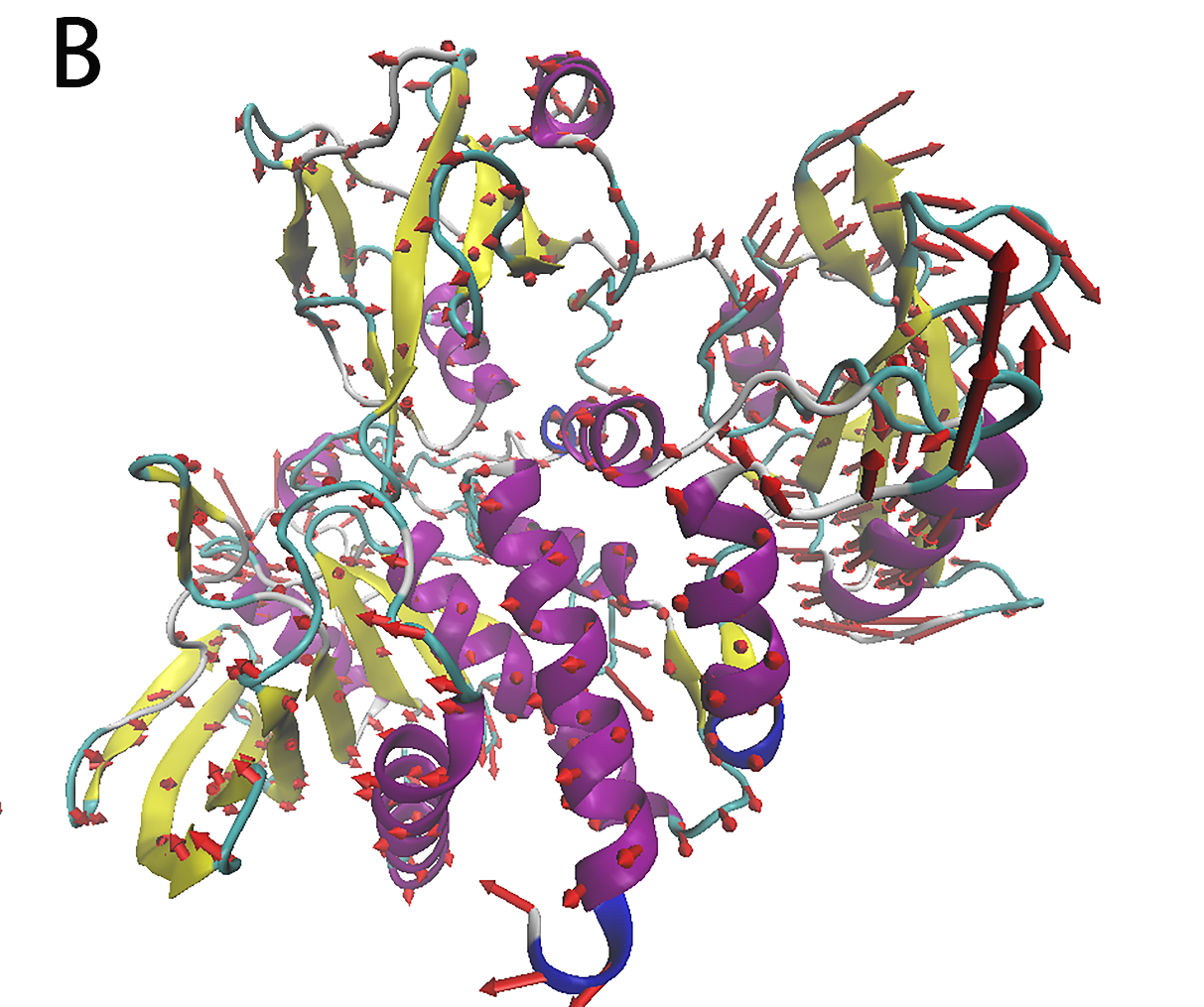


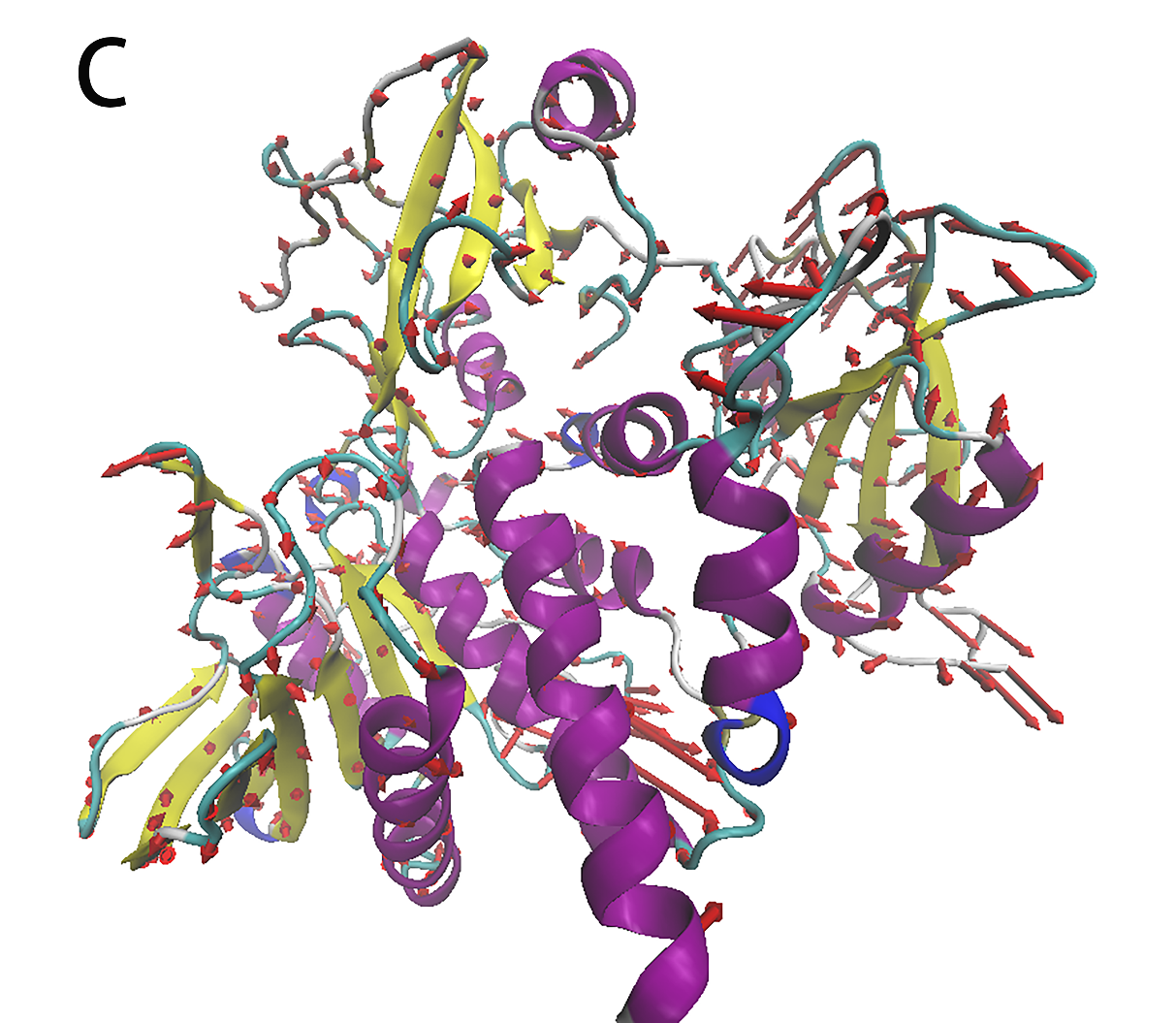


Figure S3. The cross-correlation maps of systems N-SH2-ITIM (A), N-SH2-ITSM (B), C-SH2-ITIM (C) and C-SH2-ITSM (D).


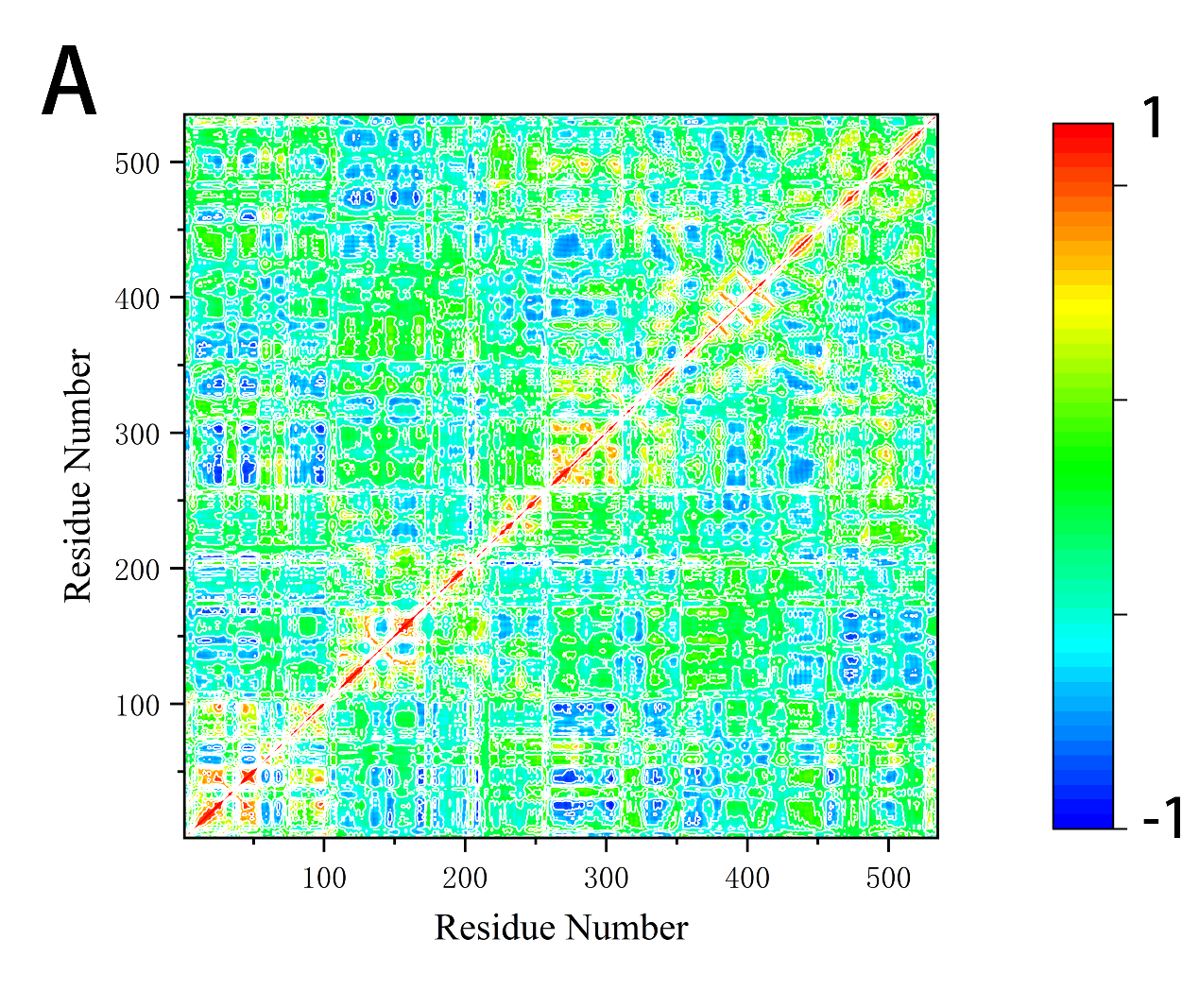


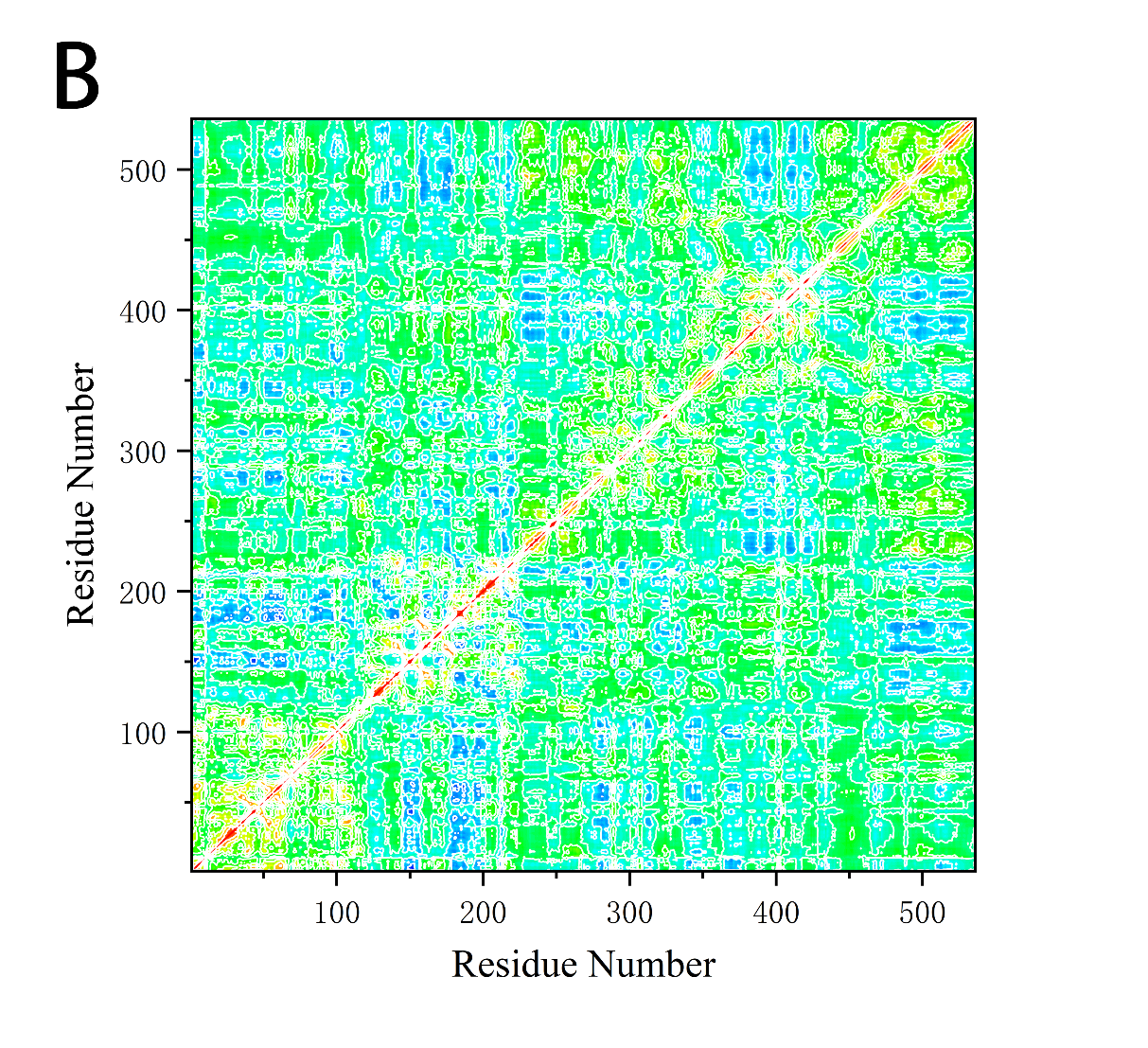

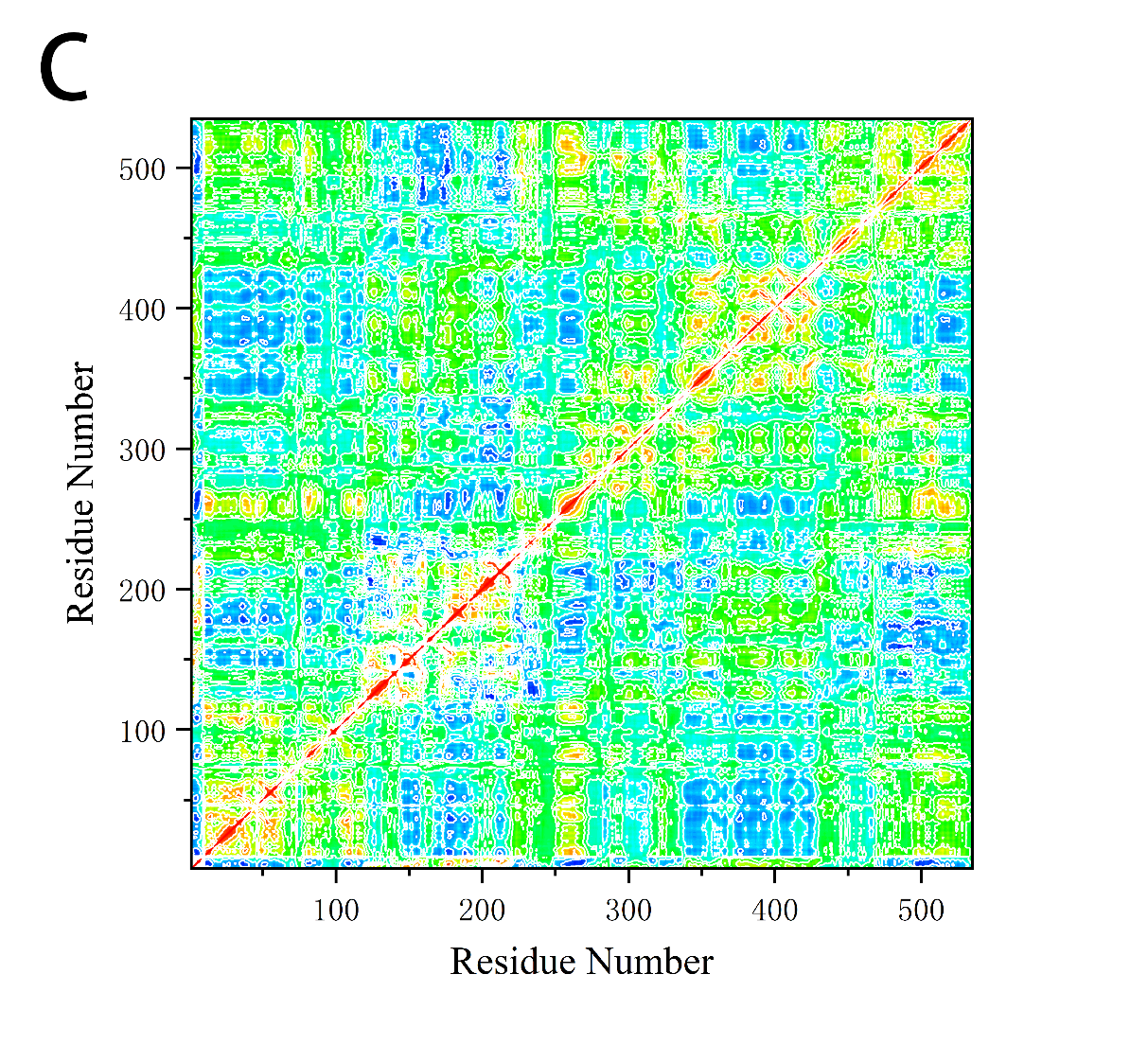

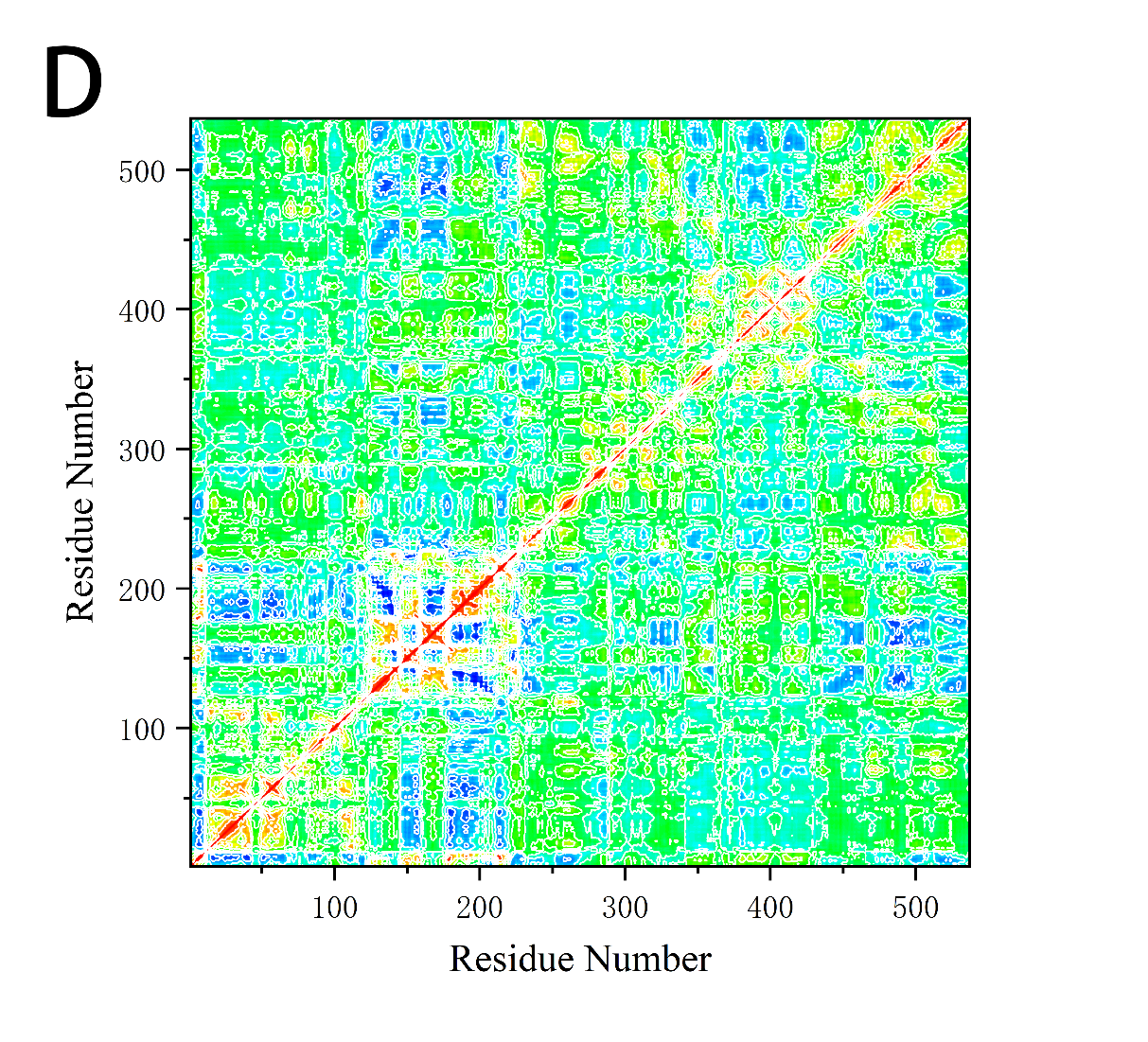


Figure S4. The FEL maps of systems N-SH2-ITIM (A), C-SH2-ITIM (B), N-SH2-ITSM (C), and C-SH2-ITSM (D).





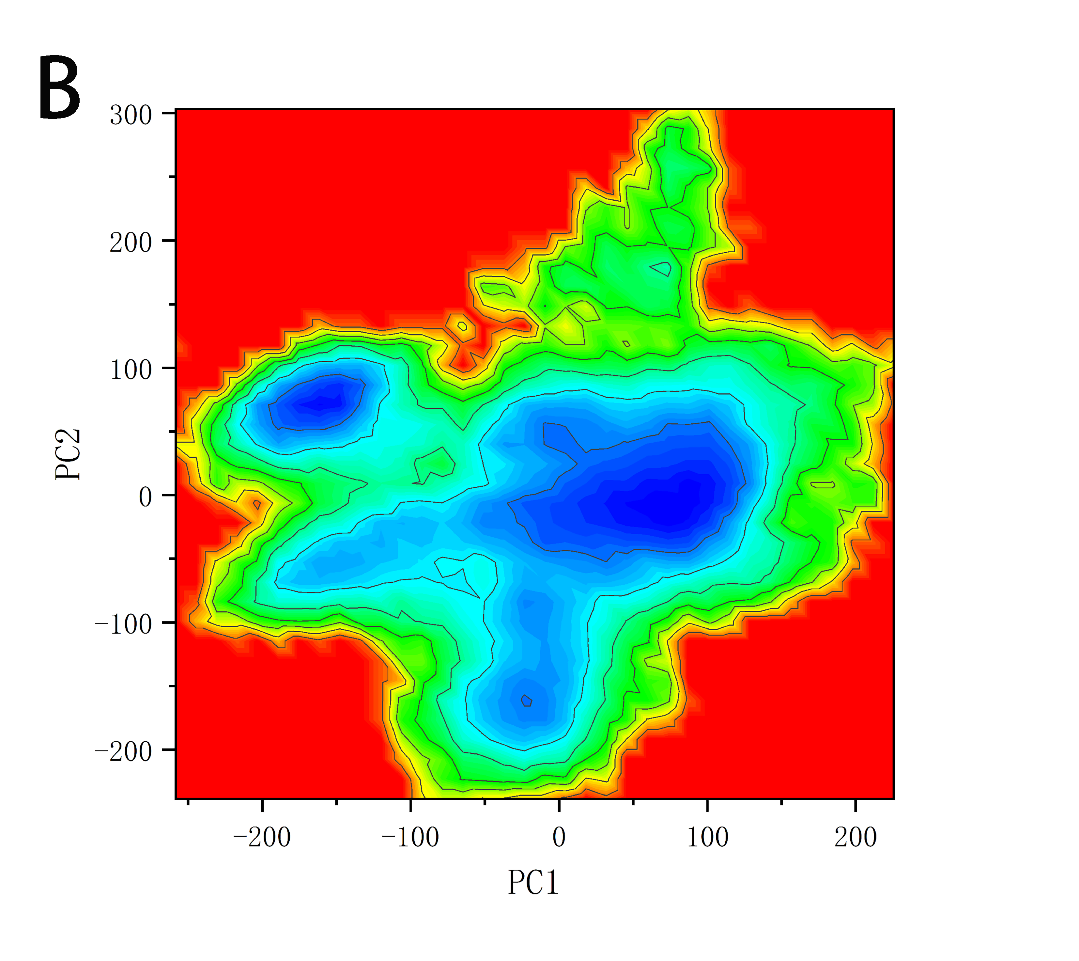


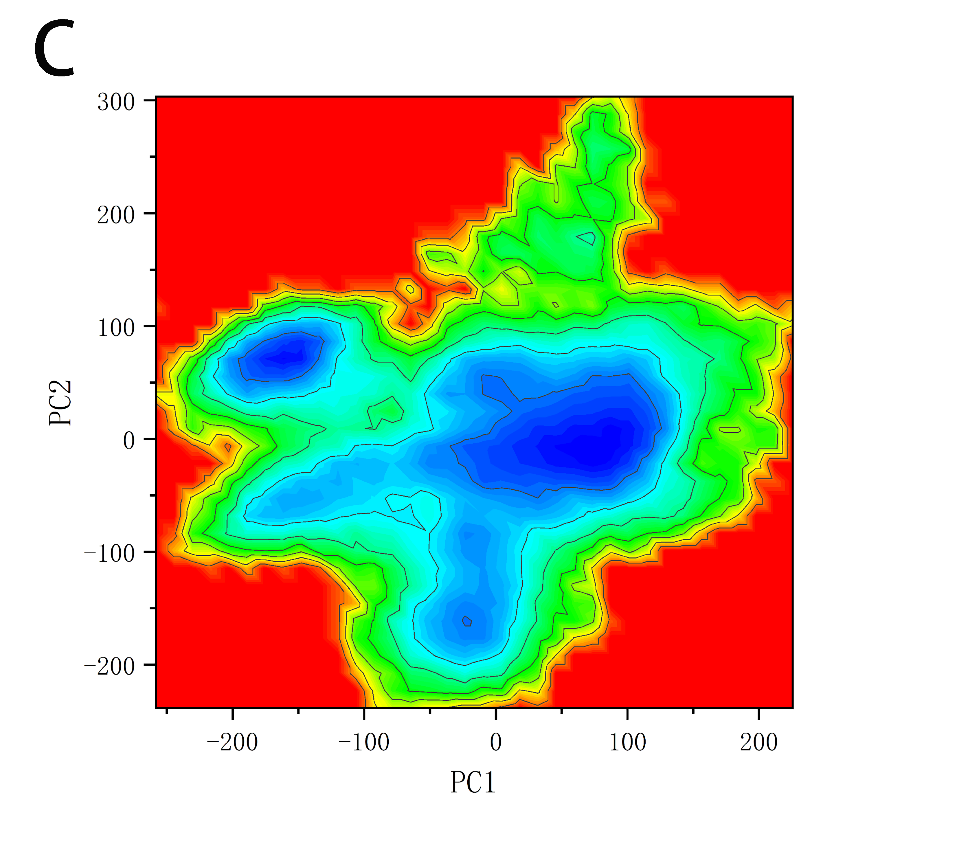


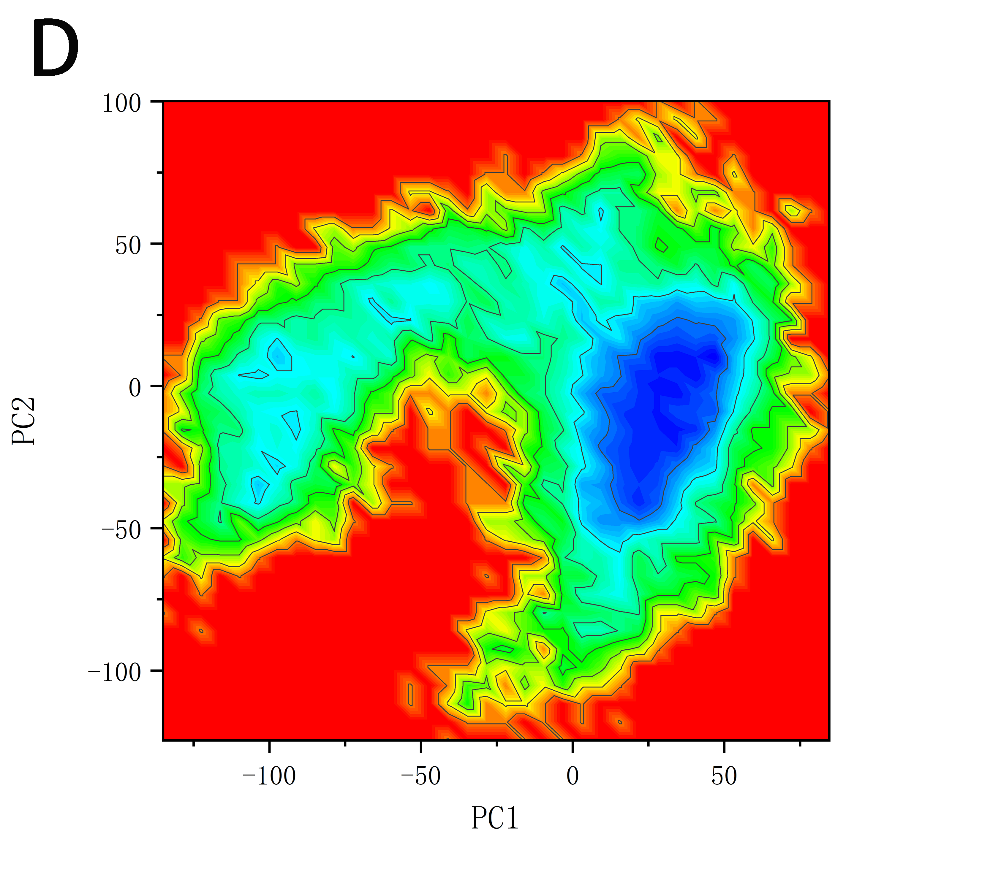


**Table S1.** Decomposition of binding energy (kcal mol^‑1^) of key residues for N-SH2-ITIM

| Residues | | ΔG_vdW_ | ΔG_ele_ | ΔG_GB_ | ΔG_SA_ | ΔG_bind_ |
| --- | --- | --- | --- | --- | --- | --- |
| ARG32 | 0.79±0.84 | | -90.57±2.46 | 76.33±1.75 | -0.10±0.02 | -13.55±1.23 |
| SER34 | | 0.33±0.86 | -18.73±1.63 | 11.33±0.84 | -0.01±0.01 | -7.07±1.04 |
| SER36 | | 0.19±0.95 | -18.00±1.40 | 11.75±0.81 | -0.23±005 | -6.30±0.78 |
| LYS91 | | -2.96± | -125.85±20.38 | 123.45±18.45 | -0.82±0.17 | -6.16±2.58 |
| LYS35 | | -0.76±0.45 | -65.96±3.04 | 61.21±2.82 | -0.05±0.01 | -5.57±0.67 |
| LYS55 | | -2.52±0.33 | -88.44±4.37 | 86.20±4.09 | -0.32±0.03 | -5.08±126 |
| HIS53 | | -2.48±0.65 | -1.67±1.17 | 1.25±0.89 | -0.47±0.07 | -3.37±0.77 |
| LEU65 | | -1.55±0.59 | -1.82±0.69 | 0.74±0.54 | -0.22±0.03 | -2.84±0.65 |
| LYS89 | | -1.94±0.50 | -59.42±6.23 | 59.09±6.08 | -0.37±0.05 | -2.64±0.58 |
| ILE54 | | -2.74±0.43 | -1.84±0.52 | 2.35±0.42 | -0.34±0.07 | -2.57±0.52 |

**Table S2.** Decomposition of binding energy (kcal mol^‑1^) of key residues for N-SH2-ITSM

| Residues | ΔG_vdW_ | | ΔG_ele_ | ΔG_GB_ | ΔG_SA_ | ΔG_bind_ |
| --- | --- | --- | --- | --- | --- | --- |
| ARG32 | | 0.72±0.92 | -80.35±2.71 | 66.33±2.01 | -0.06±0.01 | -13.35±1.40 |
| LYS55 | -1.75±0.78 | | -93.22±5.84 | 84.07±4.20 | -0.33±0.03 | -11.23±2.96 |
| HIS53 | -4.40±0.58 | | 1.78±1.30 | -3.14±0.97 | -0.51±0.03 | -6.26±1.03 |
| GLU90 | -3.51±0.57 | | 31.57±2.54 | -33.51±2.46 | -0.31±0.04 | -5.77±1.59 |
| SER36 | -0.62±0.69 | | -18.06±1.63 | 13.28±0.87 | -0.18±0.03 | -5.57±1.22 |
| LYS35 | -0.50±0.40 | | -50.03±2.50 | 45.03±2.19 | -0.01±0.01 | -5.51±0.87 |
| GLU17 | -0.79±0.86 | | -0.68±3.03 | -3.58±2.50 | -0.30±0.02 | -5.35±1.41 |
| SER34 | 0.34±0.84 | | -18.37±1.46 | 12.92±0.86 | -0.02±0.01 | -5.12±1.01 |
| THR42 | -0.57±0.64 | | -8.13±1.83 | 6.02±0.93 | -0.02±0.01 | -2.70±0.85 |

**Table S3.** Decomposition of binding energy (kcal mol^‑1^) of key residues for C-SH2-ITIM

| Residues | ΔG_vdW_ | | ΔG_ele_ | ΔG_GB_ | ΔG_SA_ | ΔG_bind_ |
| --- | --- | --- | --- | --- | --- | --- |
| ARG138 | | 0.69±0.99 | -93.32±2.81 | 80.58±2.33 | 0.10±0.02 | -12.14±1.45 |
| ARG173 | 0.56±0.87 | | -101.38±3.62 | 93.50±2.74 | -0.24±0.02 | -7.56±1.10 |
| SER140 | -0.15±0.90 | | -17.75±2.36 | 11.69±1.04 | -0.02±0.01 | -6.23±143 |
| SER142 | -0.15±0.97 | | -17.30±2.09 | 11.71±1.01 | -0.32±0.06 | -6.07±1.16 |
| GLN141 | -0.79±0.51 | | -10.60±3.20 | 6.48±2.78 | -0.05±0.02 | -4.96±0.77 |
| VAL170 | -2.74±0.37 | | -4.62±0.58 | 3.72±0.38 | -0.32±0.05 | -3.95±0.54 |
| VAL203 | -2.14±0.61 | | -1.41±0.92 | 0.93±0.65 | -0.41±0.09 | -3.02±0.76 |
| VAL148 | -1.33±0.28 | | -2.39±0.36 | 1.74±0.31 | -0.10±0.02 | -2.08±0.36 |

**Table S4.** Decomposition of binding energy (kcal mol^‑1^) of key residues for C-SH2-ITSM

| Residues | ΔG_vdW_ | | ΔG_ele_ | ΔG_GB_ | ΔG_SA_ | ΔG_bind_ |
| --- | --- | --- | --- | --- | --- | --- |
| ARG138 | | 0.27±0.92 | -101.14±4.91 | 86.19±4.15 | -0.12±0.02 | -14.80±1.84 |
| ARG186 | 0.50±0.85 | | -77.11±5.03 | 68.57±2.94 | -0.18±0.03 | -8.22±1.92 |
| SER142 | -0.26±0.89 | | -18.12±2.00 | 12.49±1.18 | -0.27±0.06 | -6.17±1.15 |
| SER140 | -0.50±0.75 | | -15.95±2.12 | 11.63±1.11 | -0.02±0.01 | -4.84±1.17 |
| GLN141 | -0.58±0.48 | | -3.90±2.34 | 0.67±1.43 | -0.04±0.03 | -3.85±1.11 |
| HIS169 | -2.46±0.55 | | -2.73±1.30 | 2.12±1.21 | -0.48±0.03 | -3.54±0.52 |
| VAL203 | -2.51±0.42 | | -2.18±0.47 | 2.07±0.35 | -0.44±0.04 | -3.06±0.57 |
| GLU204 | -2.73±0.40 | | 37.18±1.20 | -37.01±1.19 | -0.28±0.03 | -2.93±0.47 |
| GLY183 | -1.66±0.32 | | -6.21±0.69 | 5.42±0.48 | -0.17±0.03 | -2.61±0.53 |
| VAL170 | -2.27±0.32 | | -0.89±0.51 | 0.87±0.45 | -0.20±0.03 | -2.50±0.41 |
| MET171 | -1.93±0.32 | | -3.88±0.89 | 3.55±0.68 | -0.23±0.03 | -2.49±0.54 |
| THR205 | -2.05±0.45 | | -2.46±0.61 | 2.69±0.72 | -0.46±0.04 | -2.28±0.56 |

**Table S5.** Decomposition of binding energy (kcal mol^‑1^) of key residues for dual system (ITIM)

| Residues | ΔG_vdW_ | | ΔG_ele_ | ΔG_GB_ | ΔG_SA_ | ΔG_bind_ |
| --- | --- | --- | --- | --- | --- | --- |
| ARG32 | | 0.49±0.86 | -104.44±2.97 | 91.80±2.44 | -0.11±0.03 | -12.30±1.48 |
| LYS55 | -2.37±0.76 | | -110.47±13.33 | 105.73±11.01 | -0.46±0.05 | -7.56±3.79 |
| LYS35 | -0.52±0.66 | | -91.41±19.11 | 86.54±16.46 | -0.12±0.06 | -5.51±2.91 |
| SER34 | -0.08±0.82 | | -19.40±2.83 | 14.05±1.76 | -0.07±0.04 | -5.49±1.26 |
| LYS91 | -3.21±0.89 | | -101.34±8.24 | 99.84±7.46 | -0.68±0.08 | -5.38±1.50 |
| ILE54 | -2.96±0.45 | | -1.38±0.44 | 1.31±0.34 | -0.24±0.03 | -3.27±0.52 |
| LYS89 | -2.06±0.69 | | -66.61±12.73 | 66.30±12.73 | -0.40±0.12 | -2.77±1.84 |
| HIS53 | -2.02±0.58 | | 0.67±1.60 | -1.00±1.39 | -0.37±0.06 | -2.72±0.69 |
| GLU90 | -2.90±0.52 | | 62.58±4.48 | -61.72±4.60 | -0.24±0.06 | -2.27±1.07 |

**Table S6.** Decomposition of binding energy (kcal mol^‑1^) of key residues for dual system (ITSM)

| Residues | ΔG_vdW_ | | ΔG_ele_ | ΔG_GB_ | ΔG_SA_ | ΔG_bind_ |
| --- | --- | --- | --- | --- | --- | --- |
| ARG138 | | 0.67±0.87 | -91.21±3.86 | 78.26±3.09 | -0.08±0.02 | -12.36±1.44 |
| SER142 | -0.69±0.99 | | -19.20±2.20 | 13.12±1.23 | -0.39±0.07 | -7.15±1.14 |
| SER140 | 0.30±0.87 | | -18.26±1.85 | 11.40±0.91 | -0.01±0.01 | -6.57±1.04 |
| GLN141 | -0.77±0.48 | | -10.23±3.03 | 6.16±2.57 | -0.04±0.01 | -4.88±0.84 |
| HIS169 | -2.69±0.56 | | -5.38±1.49 | 4.61±1.40 | -0.46±0.05 | -3.93±0.60 |
| VAL203 | -2.22±0.50 | | -2.07±0.56 | 1.97±0.30 | -0.37±0.06 | -2.69±0.52 |
| GLU204 | -2.86±0.36 | | 40.96±1.98 | -40.43±1.95 | -0.27±0.03 | -2.61±0.53 |
| VAL170 | -2.18±0.37 | | -0.76±0.47 | 0.96±0.41 | -0.21±0.03 | -2.19±0.16 |
| VAL148 | -1.36±0.22 | | -1.68±0.38 | 0.98±0.34 | -0.07±0.01 | -2.13±0.29 |

**Table S7.** Properties of H-bonds between N-SH2 and PTP domain in system SHP2.

| system | acceptor ~ donor | occupied (%) |
| --- | --- | --- |
| SHP2 | ALA72@O ~ GLN506@HE | 85 |
|  | ASP61@OD1~ALA461@H | 85 |
|  | ASP61@OD1 ~ GLY464@H | 84 |
|  | GLY60@O ~ GLN510@HE | 74 |
|  | ASP61@OD2 ~GLN506@H | 62 |
|  | ASP61@OD2 ~ILE463@H | 49 |
|  | GLU76@OE2 ~ ARG265@H | 31 |

**Table S8.** Properties of H-bonds between N-SH2 and PTP domain in system DUAL.

| system | acceptor ~ donor | occupied (%) |
| --- | --- | --- |
| DUAL | GLU76@OE2~SER502@H | 97 |
|  | ALA72@O ~ GLN506@HE | 92 |
|  | GLY60@O ~ GLN510@HE | 75 |
|  | ASP61@OD1 ~ GLY464@H | 44 |
|  | ASP61@OD1~ALA461@H | 34 |
|  | ASP61@OD2 ~GLN506@H | 30 |

**Table S9** Properties of H bonds between SHP2 and ITIM in systems N-SH2-ITIM and C-SH2-ITIM

| system | acceptor ~ donor | Occupied (%) |
| --- | --- | --- |
| N-SH2-ITIM | HIS53@O ~ G1@N | 76 |
|  | Q6@NE2~LEU65@H | 70 |
|  | LYS89@O ~ D4@H | 66 |
|  | PTR0@O3P ~ SER36@HG | 61 |
|  | PTR0@O1P ~ARG32@H | 60 |
|  | PTR0@O3P~ SER34@H | 59 |
|  | E2@O15 ~ LYS91@H | 45 |
|  |  |  |
| C-SH2-ITIM | VAL203@O ~D4@N | 72 |
|  | PTR0@O ~ARG138@H | 53 |
|  | E2@OE1 ~ ARG173@H | 43 |
|  | PTR0@O1P ~SER142@H | 40 |
|  | E2@O ~THR205@H | 37 |
|  | PTR0@O2P ~SER170@H | 34 |
|  | PTR0@O2P ~GLN141@H | 30 |

**Table S10.** Properties of H bonds between SHP2 and ITSM in system N-SH2-ITSM and C-SH2-ITSM

| system | acceptor ~ donor | Occupied (%) |
| --- | --- | --- |
| N-SH2-ITSM | HIS53@O ~ A1@H | 75 |
|  | LYS89@O ~ V4@H | 70 |
|  | T2@O ~LYS91@N | 58 |
|  | GLU17@OE2~E-2@H | 49 |
|  | PTR0@O2P ~ SER34@HG | 45 |
|  | PTR0@O3P ~ARG32@H | 44 |
|  | PTR0@O2P ~ SER36@HG | 33 |
|  |  |  |
| C-SH2-ITSM | HIS169@O ~A1@H | 83 |
|  | PTR0@O1P ~SER140@H | 61 |
|  | P6@O ~ ARG186@H | 60 |
|  | VAL203@O ~V4@N | 55 |
|  | PTR0@O ~ARG138@H | 51 |
|  | T2@O ~THR205@H | 47 |
|  | PTR0@O1P ~SER142@H | 44 |
|  | PTR0@O2P ~SER170@H | 34 |
|  | PTR0@O2P ~GLN141@H | 30 |

**Table S11.** Properties of H bonds between SHP2 and phosphopeptides in system DUAL

| peptides | acceptor ~ donor | Occupied (%) |
| --- | --- | --- |
| ITIM | LYS89@O ~ D4@H | 64 |
|  | HIS53@O ~ G1@H | 61 |
|  | E2@O ~LYS91@N | 43 |
|  | GLU17@OE2~E-2@H | 49 |
|  |  |  |
| ITSM | HIS169@O ~A1@H | 70 |
|  | VAL203@O ~V4@N | 69 |
|  | T2@O ~THR250@H | 57 |
|  | PTR0@O1P ~SER142@H | 48 |
|  | PTR0@O ~ARG138@H | 44 |
|  | PTR0@O1P ~SER140@H | 40 |
